# Biochemical and genetic characterization of *Botrytis cinerea* mutants resistant to the plant-derived pesticide trans-dehydromatricaria ester

**DOI:** 10.3389/fpls.2025.1715720

**Published:** 2025-11-20

**Authors:** Gaijuan Tang, Rui Nan, Hui Dong, Yong Wang, Wenhui Guo, Yongquan Ta, Yunfei Han, Chao Zhang, Yonghong Wang

**Affiliations:** 1 Northwest Agriculture and Forestry (A&F) University, Yangling, Shaanxi, China; 2 Hybrid Rapeseed Research Center of Shaanxi Province, Yangling, Shaanxi, China; 3 College of Agriculture, Ningxia University, Yinchuan, Ningxia, China; 4 Environmental and Plant Protection Institute, Chinese Academy of Tropical Agricultural Sciences, Haikou, Hainan, China

**Keywords:** *Botrytis cinerea*, trans-dehydromatricaria ester (TDDE), drug resistance, antibacterial mechanism, molecular targets

## Abstract

*Botrytis cinerea* is one of the top ten plant pathogens worldwide, with a broad host range. In modern agricultural production, it is typically controlled using chemical agents. However, the overuse of chemical fungicides has led to environmental pollution and the development of drug-resistant strains, necessitating the search for new fungicides. Trans-dehydromatricaria ester (TDDE) not only enhances infectivity but also reduces lipid oxidation and improves osmotic regulation. Through the selection process using TDDE, seven highly resistant strains of *B. cinerea* with stable genetic traits were obtained. These resistant strains exhibited significant physiological alterations, including increased cell membrane permeability, heightened osmotic sensitivity, and perturbations in energy metabolism and lipid peroxidation. Additionally, TDDE had a negative cross-resistance with procymidone, but not with pyraclostrobin, boscalid and fluazinam. Resequencing and transcriptomic analysis identified six potential target genes, of which *Bcin15g03240* and *Bcin09g00290* are associated with membrane transport. Molecular docking revealed that *Bcin15g03240* interacts with TDDE via hydrogen bonds. These findings offer preliminary insight into the molecular targets of TDDE’s antimicrobial activity. Overall, the results provided a crucial theoretical foundation for understanding TDDE’s mode of action against *B. cinerea* and for developing green pesticides based on these targets.

## Graphical Abstract

**Figure:**
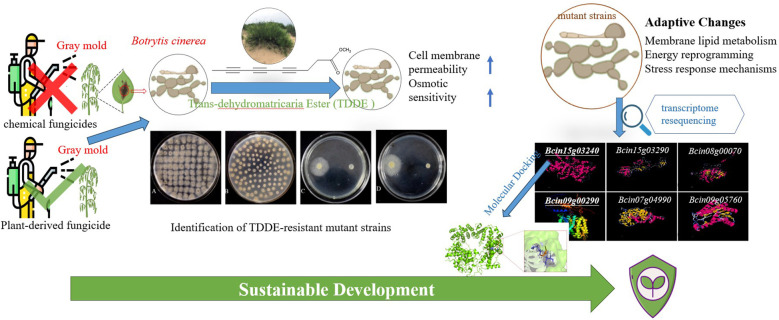
Trans-dehydromatricaria ester promotes the development of green pesticides. This is an original creation for this study.

## 1 Introduction

Gray mold, caused by *Botrytis cinerea(B. cinerea)*, is a globally devastating disease that primarily affects fruits, leading to yield losses ranging from 10% to 80% due to fruit rot (Yin et al., 2018; Mercier et al., 2019; Choquer et al., 2021). In addition to fruits, *B. cinerea* also damages leaves, stems, and flowers. This pathogen is notorious for its prolific spore production and remarkable adaptability. Among various phytopathogenic microorganisms, *B. cinerea* is recognized as a model species due to its ability to degrade plant tissues and its substantial economic impact. The Fungicide Resistance Action Committee has classified it as posing a medium to high risk of developing fungicide resistance (Corwin et al., 2016; Hou et al., 2020). The pathogen’s rapid reproduction and high adaptability have contributed to widespread resistance against several classes of fungicides, including benzimidazoles, methoxyacrylates, and succinate dehydrogenase inhibitors, thereby exacerbating the problem of multidrug resistance (Adnan et al., 2018; Cosseboom et al., 2020; Thind, 2022). Although fungicide application remains the primary strategy for managing gray mold, integrated approaches such as optimized crop management, plant protection strategies, and predictive modeling have emerged as valuable complementary tools (Kumar et al., 2023). In the area of genetic breeding, efforts have been made to develop crop varieties with resistance to *B. cinerea* (Nicot et al., 2002). However, to date, no major resistance genes conferring effective defense against this pathogen have been identified in any plant species. Consequently, chemical control continues to be the cornerstone of gray mold management. Yet, the persistent evolution of *B. cinerea* has led to escalating resistance to conventional fungicides, further complicating control efforts (Li et al., 2024). Plant-derived pesticides offer a promising alternative due to their unique advantages, including diverse bioactive compounds and ecological compatibility (Enyiukwu et al., 2014). The effectiveness of such botanical pesticides largely depends on the identification of plant species that are both resource-rich and biologically active. While compounds like toosendanin and matrine have shown considerable potential, their limited availability remains a significant bottleneck for broader application.

*Artemisia ordosica*, a wild plant with multiple potential applications, contains the bioactive compound TDDE, which has demonstrated broad-spectrum antifungal activity and holds promise as a novel fungicide. TDDE, a polyacetylene with notable biological activity, exhibits inhibitory effects not only against a range of fungi and bacteria but also against certain cancer cells, while also possessing sensitizing and anti-inflammatory properties (Greger, 1978; Tang et al., 2021; Thind, 2022). Recent studies have confirmed the potent antimicrobial efficacy of TDDE, highlighting its potential for the control of *B. cinerea*. Treatment with TDDE significantly compromised the membrane integrity and permeability of *B. cinerea*. Furthermore, structural damage and functional disruption in fungal hyphae markedly reduced the pathogen’s infectivity toward host plants. Through these mechanisms, TDDE exhibited control efficacy against gray mold, reaching 84.11% in tomatoes and 96.37% in strawberries (Tang et al., 2021). As this is the first reported study on the use of TDDE against gray mold, the compound emerges as a highly promising candidate for the development of novel and effective fungicides.

In the context of disease management, the discovery of novel fungicides is often accompanied by the inevitable emergence of resistance, posing a persistent challenge. Therefore, assessing the resistance risk of *B. cinerea* to TDDE is critical, especially given the limited current understanding of the resistance mechanisms associated with this compound. In this study, seven *B. cinerea* strains with increased resistance to TDDE were successfully induced. Among them, four representative strains were selected for biochemical and genetic analyses to evaluate their resistance risk and to preliminarily investigate the underlying resistance mechanisms. The findings from this research offer new insights into the molecular and physiological basis of TDDE resistance in *B. cinerea*, providing a foundation for future studies aimed at the sustainable application of this promising compound.

## 2 Materials and methods

### 2.1 Materials

Tomato seedlings of the cultivar Maofang 802 and the wild-type *B. cinerea* strain B05.10 were obtained from the Shaanxi Provincial Biological Pesticide Engineering Technology Research Center. The *B. cinerea* B05.10 strain was maintained on potato dextrose agar (PDA) medium at 4 °C for routine cultivation. The aerial parts of *Artemisia* ordosica were collected at the seed maturity stage in November 2017 from Shenmu Town, Yulin City, China, and were botanically identified by the College of Life Sciences, Northwest A&F University. The plant material (fresh weight: 100 kg) was air-dried to obtain 60 kg of dried powder for the extraction of active compounds. The dried powder of *Artemisia ordosica* was extracted with 80% ethanol at room temperature until the extract was nearly colorless. The combined ethanol extract was concentrated to obtain a crude extract. The concentrated extract was then further extracted with petroleum ether. The extract was separated by silica gel column chromatography (petroleum ether/acetone) to yield 11 fractions (H1-H11). Fraction H3 was purified by silica gel column (n-pentane/petroleum ether) and gel filtration. Finally, compound TDDE (purity: 95%) was obtained as the test substance. For cross-resistance assays, the following commercial fungicides were employed: fluazinam (98% technical grade; Jiangsu Ruibang, Nantong, China), boscalid (97% technical grade; Guangdong Guangkang, Guangzhou, China), and procymidone (98% technical grade; Jiangsu Jinghong, Yancheng, China). An additional batch of boscalid (97% technical grade; Guangdong Guangkang, Guangzhou, China) was included to ensure consistency and redundancy in testing.

### 2.2 Parental strain sensitivity assessment

The sensitivity of the parental strain *B. cinerea* to TDDE and four commercial fungicides—procymidone, pyraclostrobin, boscalid, and fluazinam—was evaluated using the mycelial growth rate method. Mycelial plugs were transferred to PDA plates supplemented with different concentrations of each fungicide. The tested concentrations were as follows: TDDE: 0, 0.3125, 0.625, 1.250, 2.500, and 5.000 μg/mL. The plate layout was completely randomized. Each treatment included three technical replicates. Fluazinam: 0, 0.0156, 0.0313, 0.0625, 0.125, and 0.250 μg/mL. Procymidone: 0, 0.078, 0.156, 0.313, 0.625, and 1.250 μg/mL. Boscalid: 0, 0.313, 0.625, 1.250, 2.500, and 5.000 μg/mL. Pyraclostrobin: 0, 0.625, 1.250, 2.500, 5.000, and 10.000 μg/mL. Treated plates were incubated at 25–28 °C for 3–5 days (Wang et al., 2016), after which the colony diameters were measured. The mycelial growth inhibition rate was calculated using (Equation 1). The half-maximal effective concentration (EC_50_) for each fungicide was determined by probit analysis using SPSS software (version 26.0) (Wang et al., 2017b). The mean EC_50_ value was used for further analysis. This experiment aimed to evaluate the antifungal efficacy of TDDE in comparison with commonly used fungicides against *B. cinerea.*

(1) $$\begin{array}{c} \text{Mycelial growth inhibition rate}\left(\%\right)=\left(\text{control colony diameter}-\text{treated colony diameter}\right)\\ /\left(\text{control colony diameter}–6\right)\times 100\% \end{array}$$

### 2.3 Induction of TDDE-resistant mutants of *B. cinerea*

The parental strain was cultured on PDA medium until the mycelium fully covered the plate surface. Mycelial plugs (6 mm in diameter) were excised from the colony margin using a sterile punch and transferred, mycelial side up, onto PDA plates supplemented with 5 μg/mL TDDE. Once the mycelium grew to cover approximately two-thirds of the plate, it was subcultured onto TDDE-free PDA. When the colony again reached two-thirds coverage, it was transferred back onto PDA containing 5 μg/mL TDDE. This induction-recovery cycle was repeated at least three times. Over a period of 24 months, the parental *B. cinerea* strain was subjected to gradually increasing TDDE concentrations (10, 20, 40, 80, 160, 200, 300, and 400 μg/mL) to induce resistant mutants. To ensure the universality of the resistance mechanisms and to investigate their diversity, this study selected seven mutant strains with significantly elevated EC_50_ values as subjects for further investigation.

### 2.4 Stability testing of TDDE-resistant mutant strains

To evaluate the stability of TDDE resistance in the selected strains, the isolates were subcultured on TDDE-free PDA medium for 10 consecutive generations. The EC_50_ values were determined for both the first and tenth generations to assess any changes in resistance. Additionally, the strains were stored at 4 °C for 60 days to further examine the persistence of resistance. The experiments were performed with three biological replicates and repeated independently three times.

### 2.5 Determination of mycelial growth, mycelial morphology, and sporulation

Mycelial fragments from 3- to 5-day-old colonies were transferred onto PDA plates supplemented with fungicides and incubated at 25 °C for 3 days. The colony diameters of each strain were measured, and mycelial morphology was observed under a microscope. Both parental and resistant strains were inoculated on PDA and cultured for 4 days. The resulting mycelium was harvested, dried, and weighed to determine the mycelial biomass. For sporulation assessment, the strains were point-inoculated on PDA plates and incubated at 25 °C under a 12 h/12 h light/dark cycle for 7 days to promote conidiation. Following incubation, spores were harvested by gently scraping the colony surface with 10 mL of sterile water containing 0.05% (v/v) Tween-80. The resulting spore suspension was filtered through four layers of sterile cheesecloth to remove mycelial debris. Spore concentration was determined using a hemocytometer under a light microscope, and expressed as spores per milliliter.

### 2.6 Cross-resistance

The sensitivity of TDDE-resistant and parental strains to TDDE, fluazinam, procymidone, pyraclostrobin, and boscalid was assessed by calculating their EC_50_ values, using the same experimental procedures described in Section 2.2. Spearman’s rank correlation analysis was performed to evaluate cross-resistance between TDDE and the other four fungicides. According to the correlation coefficient (ρ), cross-resistance was categorized as follows: no cross-resistance (ρ > 0.05 or ρ < 0.1), significant cross-resistance (0.8 < ρ ≤ 1 and ρ < 0.05), strong cross-resistance (0.6 ≤ ρ ≤ 0.8), moderate cross-resistance (0.4 ≤ ρ < 0.6), low cross-resistance (0.2 ≤ ρ < 0.4), and very low cross-resistance (0.1 ≤ ρ < 0.2) (Li X. H., 2021).

### 2.7 Growth of TDDE-resistant mutant strains under different pH, temperature, and light conditions

Different pH: The pH of PDA medium was adjusted to five gradients (5, 6, 7, 8, and 9) using 1 mol/L HCl and NaOH solutions. The parental strain and resistant strains were inoculated on media with different pH levels and cultured at 25 °C for 3 days. Colony diameters were measured using the cross method. Each treatment was repeated three times.

Different temperatures: The parental strain and resistant strains were inoculated on medium and subjected to four temperature treatments (5, 15, 25, and 30 °C). After 3 days of culture, colony diameters were measured using the cross method. Each treatment was repeated three times.

Different light durations: The parental strain and resistant strains were inoculated on medium and exposed to three light treatments (0, 12, and 24 hours of light). Cultures were maintained at 25 °C for 3 days, and colony diameters were measured using the cross method. Each treatment was repeated three times.

### 2.8 Pathogenicity assessment

To ensure that the TDDE resistance would be maintained throughout the subsequent experiments, a robust selection strategy was employed. From the seven mutant strains, we specifically selected the two that exhibited a further increase in resistance and the two that showed the smallest decrease, as these strains represented the phenotypes most likely to sustain stable resistance over the long term. These four strains were therefore used for all pathogenicity assessments and subsequent experiments. *In vitro* detached leaf assay: Tomato and strawberry leaves were rinsed under running water and surface-sterilized with 75% ethanol. A 6 mm diameter wound was made on the leaf surface using a sterile 1 mL syringe, and an inverted 6 mm mycelial plug (mycelium side facing downward) was placed onto the wound using the same syringe. Each treatment included three biological replicates. Each leaf was wrapped with plastic film to secure the plug and prevent detachment. Inoculated leaves were incubated under humid conditions at 25 °C for approximately 3 days. Lesion areas were measured using ImageJ software (https://imagej.net/ij/), and the relative lesion area was calculated. Outdoor potted plant assay: This assay was conducted with three biological replicates. Tomato seedlings with uniform growth at the four-leaf stage were selected for inoculation, following the same procedure described in the detached leaf assay (Wang et al., 2017a).

### 2.9 Determination of physiological indicators

Oxalic Acid Content and Malondialdehyde (MDA) content. A standard curve for oxalic acid quantification was established by adding different volumes of sodium oxalate solution to centrifuge tubes containing HCl-KCl buffer, FeCl_3_, and sulfosalicylic acid. The absorbance of each mixture was measured at 510 nm and plotted against the sodium oxalate concentration. For sample analysis, mycelial cultures grown in PDB medium for three days were filtered, and the resulting filtrates were centrifuged to obtain the supernatant. These samples were treated following the same procedure used for the standard curve, and absorbance values were recorded. Oxalic acid concentrations were calculated based on the standard curve equation. MDA content was determined using a commercial detection kit (product No.: BC0020) (Cao et al., 2024).

Glycerol content. Mycelial disks (6 mm in diameter) were excised from the margins of 3-day-old colonies and inoculated into 250 mL flasks containing 100 mL of PDB medium, with ten disks per flask. All treatments were performed in triplicate. The cultures were then fermented for an additional 3 days under the same conditions. After incubation, mycelia were filtered, gently blotted dry with absorbent paper, and rapidly frozen in liquid nitrogen. Glycerol content was measured using a commercial glycerol detection kit.

Relative Conductivity. Mycelial fragments from 3-day-old resistant strains were cultured in PDB medium for 48 hours. After filtration, washing, and weighing, 500 mg of fresh mycelia were suspended in sterile water for various durations. Conductivity was measured at 0, 5, 15, 45, 75, and 135 minutes, followed by boiling the samples for 5 minutes. Relative conductivity was calculated as the ratio of conductivity at each time point to the final (boiled) conductivity (Wang et al., 2017a).

NaCl Sensitivity Assay. Osmotic and membrane stress tolerance were evaluated by supplementing PDA medium with 0.5%, 1.5%, and 2% NaCl. Mycelial plugs were inoculated onto the medium, and colony diameters were measured after 3 days of incubation at 25 °C. Drug-free PDA plates were used as controls. The inhibition rate of mycelial growth was calculated using (Equation 1). All treatments were conducted in triplicate.

Determination of Total Sugar, Protein, ATP Content, and Na^+^/K^+^-ATPase Activity. Mycelial cultures grown for three days in PDB were harvested by vacuum filtration. The collected mycelia were ground into a fine powder under liquid nitrogen. A 0.1 g aliquot of the powdered sample was homogenized in 1 mL of extraction buffer in an ice bath. The homogenate was centrifuged at 8000 ×g at 4 °C for 10 minutes, and the supernatant was collected for analysis. Total sugar, protein, ATP content, and Na^+^/K^+^-ATPase activity were measured using the following commercial kits: Total Sugar Content Detection Kit (item No.: BC2710), Biuret Protein Content Detection Kit (item No.: BC3180), ATP Assay Kit, and Na^+^/K^+^-ATPase Activity Detection Kit (provided by Jian Cheng, Nanjing).

### 2.10 Resequencing of TDDE-resistant mutant strains

DNA samples were rigorously tested for quality, including assessments of purity and concentration, before proceeding with library construction using the TruSeq kit. The qualified libraries were subsequently constructed and sequenced on an Illumina NovaSeq 6000 platform by LC-Bio Technology Co., Ltd. (Hangzhou, China). Libraries were quantified, size-checked, and sequenced on the Illumina NovaSeq 6000 platform. Raw sequencing data were filtered, aligned, deduplicated, and subjected to SNP detection under stringent parameters to ensure high-quality data for subsequent bioinformatics analysis. The raw resequencing data of the mutant strains have been deposited in the Genome Sequence Archive under accession number CRA030238.

### 2.11 Transcriptome changes before and after TDDE treatment

Mycelial plugs (6 mm in diameter) were excised from the margin of 3-day-old colonies and inverted at the center of PDA plates containing TDDE at its EC_50_ concentration. Drug-free PDA plates served as a blank control. The plates were incubated at 25 °C in a constant-temperature chamber for 3 days. After incubation, the mycelia were harvested for transcriptome sequencing. Each treatment included three biological replicates. Total RNA was isolated and purified from *B. cinerea*, with quality assessed using TRIzol, NanoDrop, Bioanalyzer, and agarose gel electrophoresis (Wang et al., 2012; Kim et al., 2015; Beekman et al., 2018). Sequencing libraries were prepared for Illumina NovaSeq 6000 platform sequencing (Florea et al., 2013; Pertea et al., 2015). The raw sequencing data were preprocessed, aligned to the genome, assembled, annotated, and quantified. Differential gene expression analysis was conducted using edgeR, and functional annotations were performed through Gene Ontology (GO) and KEGG pathways (Kanehisa et al., 2007; Young et al., 2010). RNA extraction from *B. cinerea* was performed following TDDE treatment, and cDNA synthesis along with qRT-PCR gene expression analysis was conducted in triplicate. The raw RNA-seq data generated in this study have been deposited in the Genome Sequence Archive under accession number CRA030257.

### 2.12 qRT-PCR validation

All qRT-PCR analyses in this study were performed with three independent biological replicates. Total RNA from each biological replicate was independently reverse-transcribed into cDNA using the PrimeScript™ RT reagent Kit with gDNA Eraser (Takara). The qRT-PCR reactions for each cDNA sample were performed in triplicate on a Quant Studio 3 real-time PCR system (Thermofisher) with the following program: pre-denaturation at 95 °C for 2 min; 40 cycles of denaturation at 95 °C for 15 s, and annealing/extension at 60 °C for 30 s, with fluorescence signal acquisition at this step. Melting curve analysis was automatically generated by the system to confirm amplification specificity. The relative expression levels of the target genes were calculated using the software-integrated analysis module. The sequences of the specific primers used are listed in **Supplementary Table S6**.

### 2.13 Homology modeling and molecular docking of TDDE target gene proteins

Molecular docking was employed to predict the interactions between TDDE and target gene proteins, providing valuable insights for the design of fungicidal lead compounds. High-homology target proteins were identified using the NCBI database, and 3D models were generated and evaluated using SWISS-MODEL and SAVES 5.0. Active binding sites were predicted using Molegro Virtual Docker, and these predictions were compared with blind docking results to identify optimal TDDE binding regions. Molecular docking of TDDE with target proteins was performed using AutoDockTools, with receptor and ligand preparation, Lamarckian genetic algorithm-based search parameters, and energy calculations using a semi-empirical scoring function.

### 2.14 Statistical analysis

For all experiments in this study (including mycelial growth, sporulation, physiological indicators, pathogenicity assays, and qRT-PCR analysis), data are presented as the mean ± standard deviation (SD) of at least three independent replicates. Statistical significance among different experimental groups was assessed using one-way analysis of variance (ANOVA) followed by Tukey’s *post-hoc* test for multiple comparisons. All statistical analyses were performed using SPSS 26.0, and a p-value of less than 0.05 was considered statistically significant.

## 3 Results

### 3.1 Identification of TDDE-resistant mutant strains

**Figure 1:**
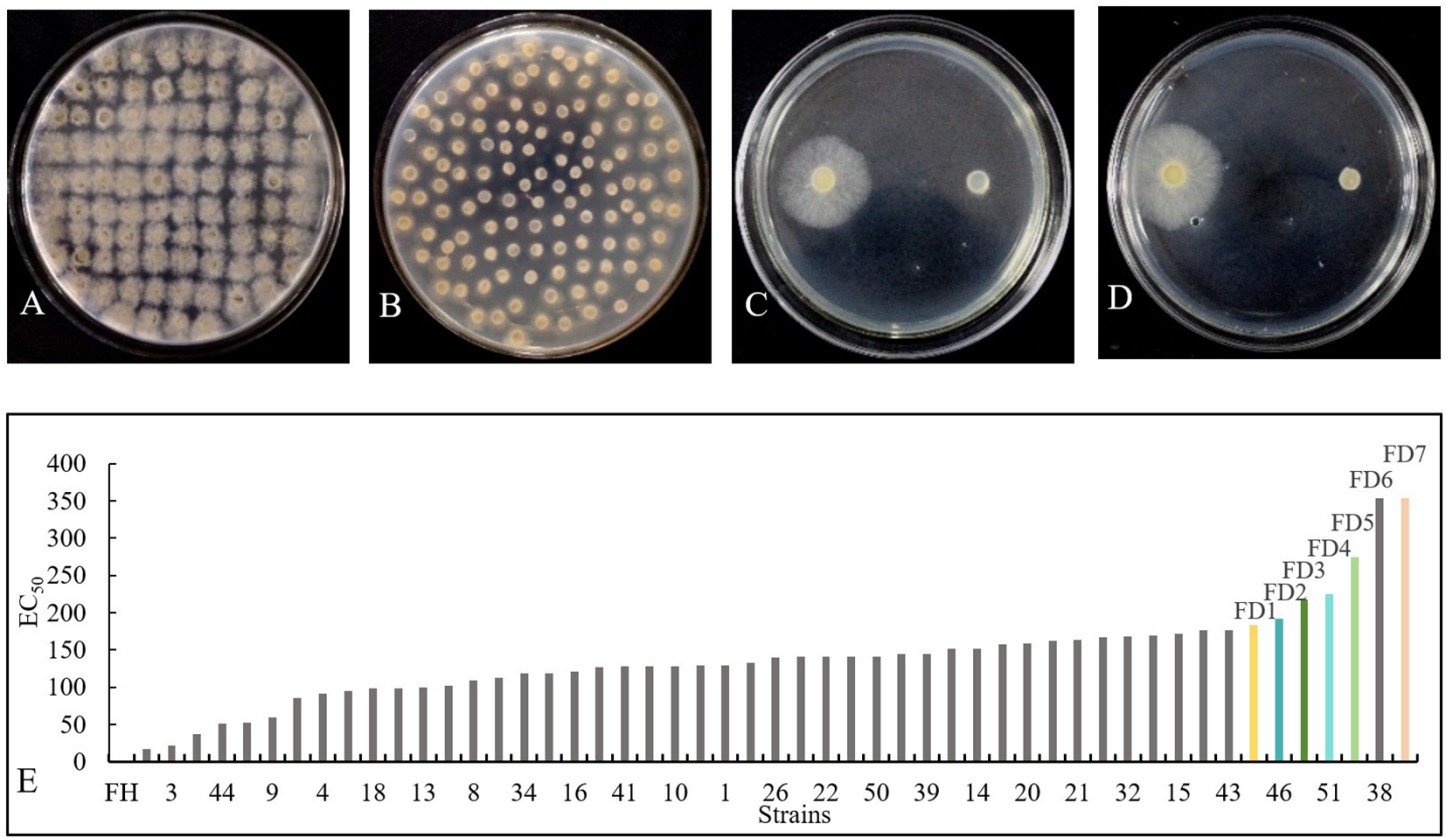
Screening of TDDE-resistant mutant strains. **(A)** The initial induction stage. **(B)** The resistant strain at a concentration of 200 μg/mL. **(C)** Comparing the effects of FD1 (left) and the parental strain (right) at a concentration of 1.401 µg/ml (EC_50_). **(D)** Comparing the effects of FD2 (left) and the parental strain (right) at a concentration of 1.401 µg/ml (EC_50_). **(E)** Virulence Determination of Different *B*. *cinerea* Strains Against TDDE. FH is the parent.

The sensitivity of the parental *B. cinerea* strain B05.10 to pyraclostrobin, boscalid, procymidone, fluazinam, and TDDE was assessed. The results showed that the parental strain exhibited no resistance to any of these fungicides at the initial testing stage. The EC_50_ values for the fungicides were determined to be 3.030, 5.146, 0.155, 0.002, and 1.401 µg/mL, respectively (**Table 1**). Subsequently, 52 highly resistant strains were successfully selected through stepwise increases in TDDE concentration up to 400 µg/mL every 5 days over the course of 100 generations (**Figure 1**). The EC_50_ values for these resistant mutants ranged from 22.227 to 354.188 µg/mL. Among them, seven strains (FD1, FD2, FD3, FD4, FD5, FD6, and FD7) exhibited the highest resistance, with EC_50_ values ranging from 183.809 to 354.188 µg/mL, and resistance ratios ranging from 132 to 253 times that of the parental strain (**Figure 1E**). According to the criteria of the Fungicide Resistance Action Committee, all strains developed in this experiment were classified as highly resistant.

**Table 1:** Sensitivity determination of *B. cinerea*.

Fungicide	Toxicity regression equation	EC_50_ (95%FL) (ug/mL)	R^2^	χ2
Pyraclostrobin	Y= 3.423-1.648X	3.030(2.567-3.546)	0.913	24.552
Boscalid	Y=1.680-1.195X	5.146(4.079-7.187)	0.918	7.493
Procymidone	Y=1.680-1.195X	0.155(0.140-0.171)	0.987	5.776
Fluazinam	Y=1.113 + 3.141X	0.002(0.001-0.004)	0.876	4.586
TDDE	Y=2.95-0.432X	1.401(1.259-1.544)	0.997	0.709

To assess the stability of resistance, the resistant strains were continuously cultured on drug-free PDA plates for 10 generations. The results indicated no significant changes in resistance levels after this prolonged culture (**Table 2**), suggesting that the resistant phenotype can be stably inherited through asexual reproduction.

**Table 2:** Determination of the genetic stability of TDDE-resistant strains.

Strain	Resistance multiple		Sensitivity change factor	Sensitivity phenotype
	Generation 1	Generation 10		
FD1	131.198	136.67	0.959962	HR
FD2	137	136.14	1.006349	HR
FD3	155.403	152.79	1.017102	HR
FD4	160.503	157.81	1.017065	HR
FD5	195.745	172.68	1.133571	HR
FD6	252.810	244.655	1.033332	HR
FD7	252.811	267.27	0.945901	HR

HR, stands for high resistance.

### 3.2 Cross-resistance and biological fitness of TDDE-resistant mutant strains

**Figure 2:**
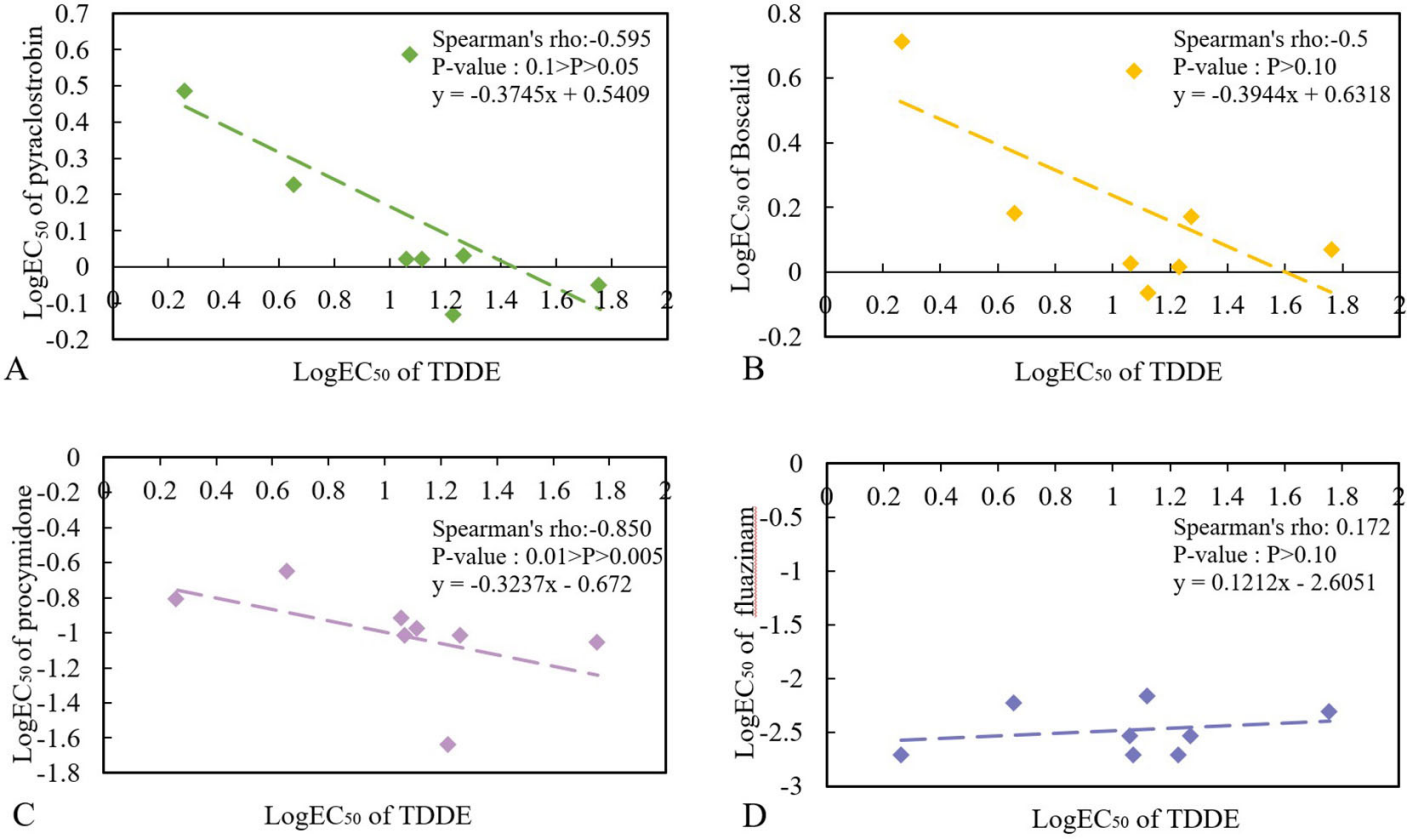
Cross-resistance determination of TDDE to pyraclostrobin **(A)**, boscalid **(B)**, procymidone **(C)**, fluazinam **(D)**.

The cross-resistance between TDDE and commonly used field fungicides was assessed. The results revealed a negative cross-resistance between TDDE and procymidone (Spearman’s ρ = -0.85, 0.01 > *P* > 0.005, n = 8). In contrast, TDDE did not exhibit any cross-resistance with pyraclostrobin (Spearman’s ρ = -0.595, 0.1 > *P* > 0.05, n = 8), boscalid (Spearman’s ρ = -0.5, P > 0.10, n = 8), or fluazinam (Spearman’s ρ = 0.172, P > 0.10, n = 8) (**Figure 2**). Therefore, it can be concluded that TDDE can be used in combination with or as an alternative to pyraclostrobin, boscalid, and fluazinam in field applications.

**Figure 3:**
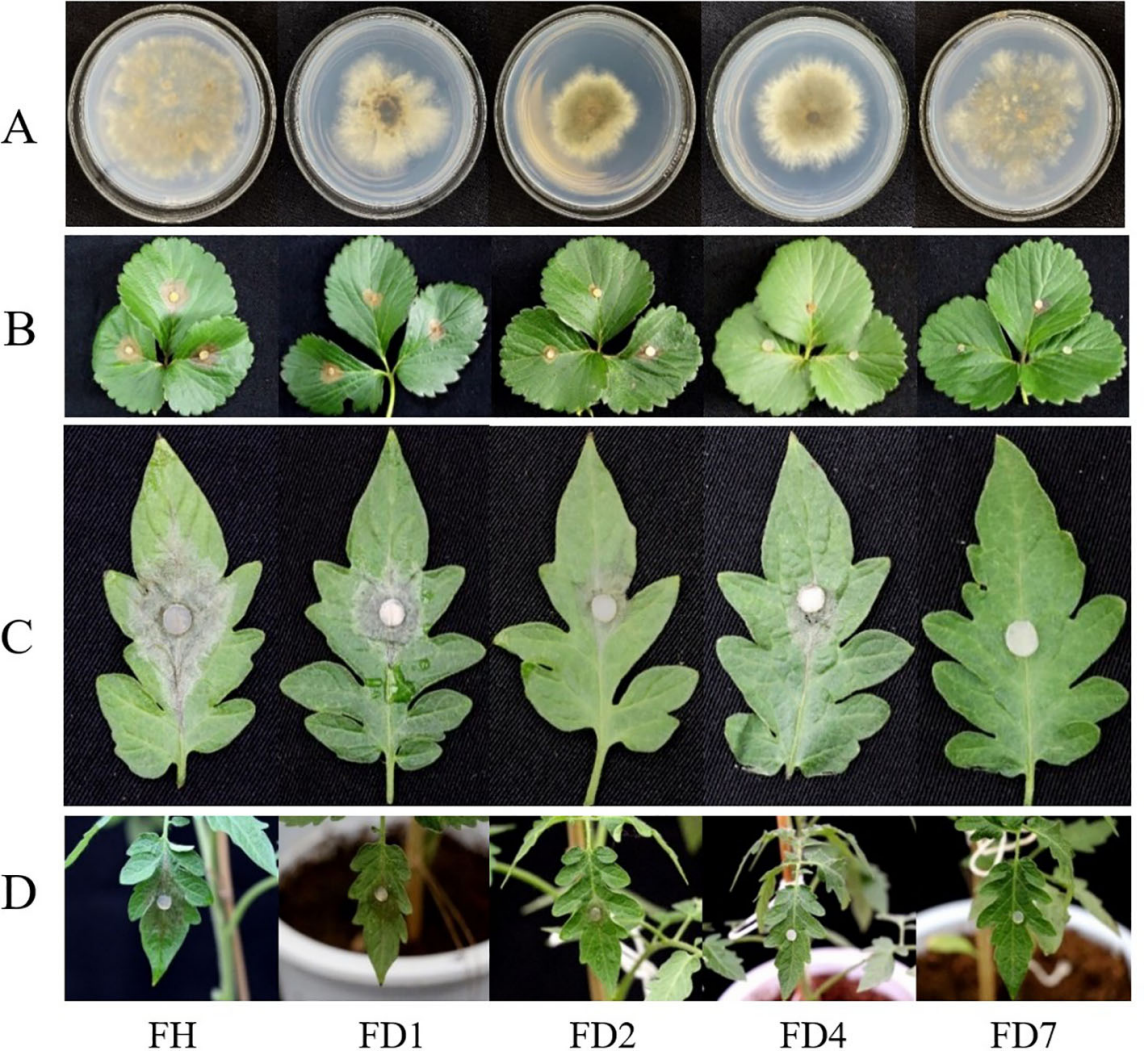
Morphology and disease incidence *in vitro* and *in vivo* of drug-resistant strains. **(A)** Strains, **(B)** Strawberry leaves, **(C)** Tomato leaves, **(D)** Tomato seedling. FH is the parent; FD1, FD2, FD4 and FD7 were resistant strains.

Under optical microscopy, the morphology of the hyphae was observed. The results showed that, compared to the parent strain, the mutant strains exhibited increased branching and aggregation of the hyphal bodies (**Supplementary Figure S1**). The stronger odor released by FD6 and FD7 suggests that their metabolites may have undergone changes (**Supplementary Figure S1**). Additionally, the mutant strains exhibited slower growth rates and reduced mycelial dry weights, with FD4 showing the most significant reduction. Sporulation varied among the mutants, with FD2 and FD4 producing more spores, while FD7 produced fewer spores than the parental strain (**Table 3**). The pathogenicity of four resistant strains (with relatively small increases or decreases in TDDE resistance) (**Table 2**) was tested using *in vitro* tissues and live tomato seedlings. The results showed that four resistant strains (FD1, FD2, FD4, and FD7) had reduced pathogenicity in both isolated leaves and tomato seedlings. Significant differences were observed in the incidence rates of these strains. Specifically, FD7 lost pathogenicity in both *in vitro* and *in vivo* assays, while FD1, FD2, and FD4 exhibited weakened pathogenicity compared to the parental strain (**Figure 3**) (**Supplementary Table S1**).

**Table 3:** Biological characteristics of strains resistant to TDDE mutations.

Strains	Sensitivity phenotype	Colony diameter(mm)	Mycelial dry weight(g)	Spore production quantity (ml^-1^)
FD1	HR	34.5 ± 0.12^b^	(0.0634 ± 0.0076)^d^	(4 ± 0.5774)×10^5c^
FD2	HR	28.3 ± 0.31^d^	(0.0814 ± 0.0203)^c^	(20.83 ± 1.5275)×10^5a^
FD3	HR	31.0 ± 0.15^c^	(0.0645 ± 0.0155)^de^	(3.8 ± 0.5701)×10^5c^
FD4	HR	22.2 ± 0.49^f^	(0.0582 ± 0.0054)^e^	(38.33 ± 2.7538)×10^5a^
FD5	HR	28.3 ± 0.36^d^	(0.0884 ± 0.0198)^bc^	(6.214 ± 0.9512)×10^5bc^
FD6	HR	28.2 ± 0.25^d^	(0.0875 ± 0.0243)^c^	(7.2 ± 1.61)×10^5b^
FD7	HR	24.3 ± 0.26^e^	(0.0938 ± 0.0291)^b^	(0.815 ± 2.46)×10^5d^
FH	S	38.0 ± 0.14^a^	(0.1501 ± 0.0617)^a^	(5.527 ± 0.3684)×10^5c^

Different lowercase letters in the figures indicate signiﬁcant differences among different strains at 0.05 level. HR stands for high resistance. S stands for sensitive.

The growth characteristics of both the parental and resistant strains were examined under varying pH, temperature, and light conditions. All strains reached their maximum colony diameter under conditions of pH 5, a temperature of 25 °C, and a 12-hour light/dark cycle (**Supplementary Figure S2**). Resistance to TDDE did not significantly alter these biological characteristics.

### 3.3 Physiological and biochemical mechanisms of TDDE-resistant mutant strains

**Figure 4:**
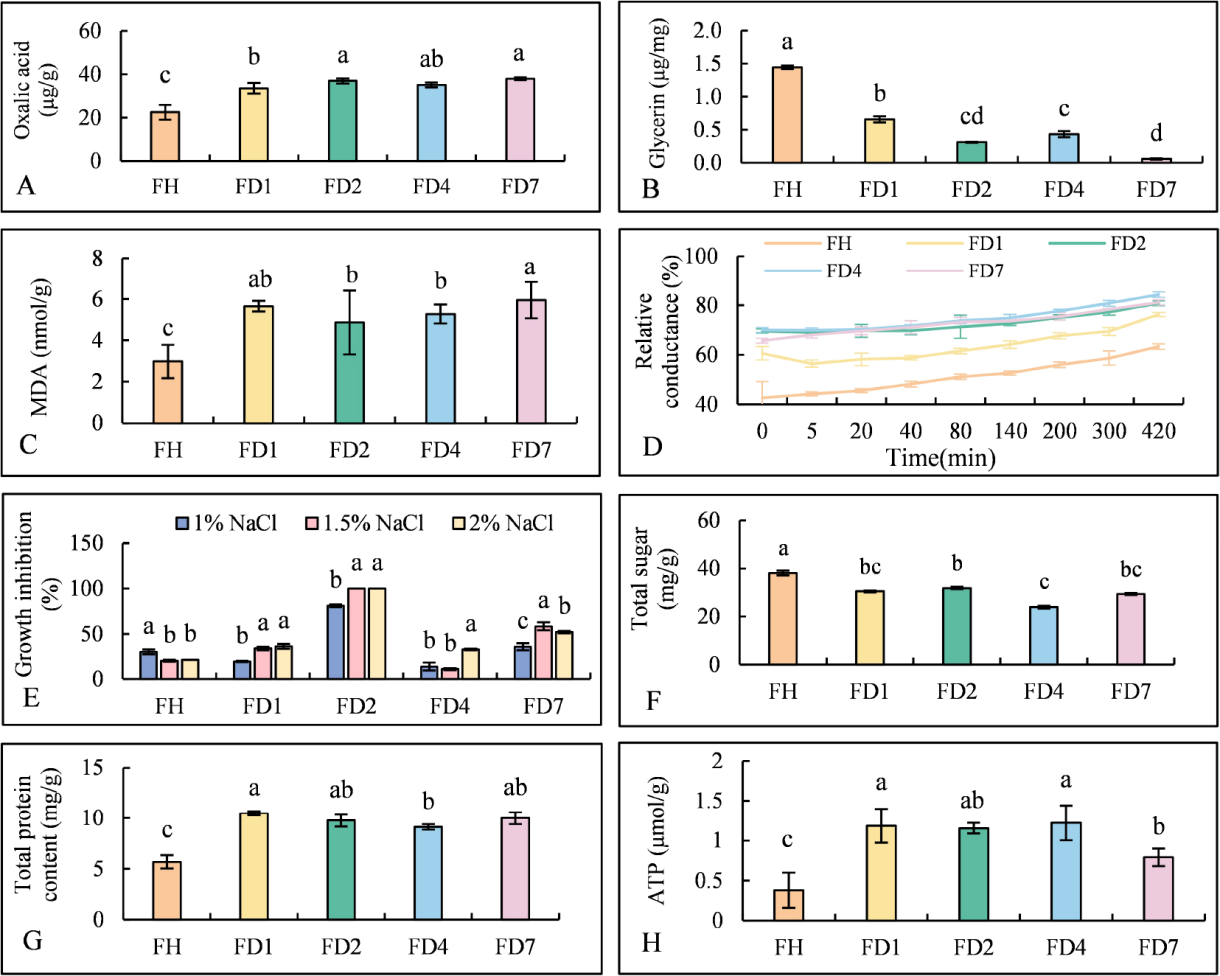
The differences in physiological characteristics between the parental of *B*. *cinerea* Strains and the drug resistant strains. **(A)** Oxalic Acid Content. **(B)** Glycerin. **(C)** MDA. **(D)** Relative Conductivity. **(E)** NaCl Sensitivity. **(F)** Total Sugar. **(G)** total protein content. **(H)** ATPase Activity. Different lowercase letters above bars indicate significant differences among treatments (p < 0.05).

The oxalic acid content, osmotic regulation, lipid peroxidation, and other physiological characteristics of the parental strain and four resistant strains were compared. The resistant strains exhibited elevated oxalic acid levels and reduced glycerol content compared to the parental strain, suggesting compromised osmotic regulation (**Figures 4A, B**). Increased malondialdehyde (MDA) levels indicated enhanced lipid peroxidation and potential membrane or mitochondrial dysfunction in the resistant strains (**Figure 4C**). All resistant strains showed higher relative conductivity and increased sensitivity to osmotic pressure, with some strains exhibiting greater growth inhibition under NaCl stress (**Figures 4D, E**). Additionally, the resistant strains exhibited metabolic shifts characterized by decreased total sugar content, increased protein and ATP levels, and elevated ATPase activity (**Figures 4F–H**), collectively indicating altered energy metabolism and potential modifications in transport mechanisms. These physiological and biochemical adaptations, alongside changes in osmotic regulation and oxidative stress responses, delineate a comprehensive defense profile associated with TDDE resistance (**Figure 4**). In conclusion, TDDE resistance was associated with significant changes in osmotic regulation, oxidative stress responses, and energy metabolism.

### 3.4 Resequencing of TDDE-resistant mutant strains

The mutant strains were resequenced to identify genetic variation loci. Resequencing data were analyzed using Illumina sequencing, with Q scores used to assess data accuracy. The AQ30 score (error probability of 0.001) indicated high-quality base sequencing, and the average Q20 proportion exceeded 95%, with Q30 above 90% for FD1, FD2, FD4, and FD7, confirming the high quality of the sequencing data. Coverage and depth analysis revealed that when the base coverage of all four mutant strains reached 96×, the average coverage depth exceeded 10%, indicating that the detected mutations are relatively reliable.

**Figure 5:**
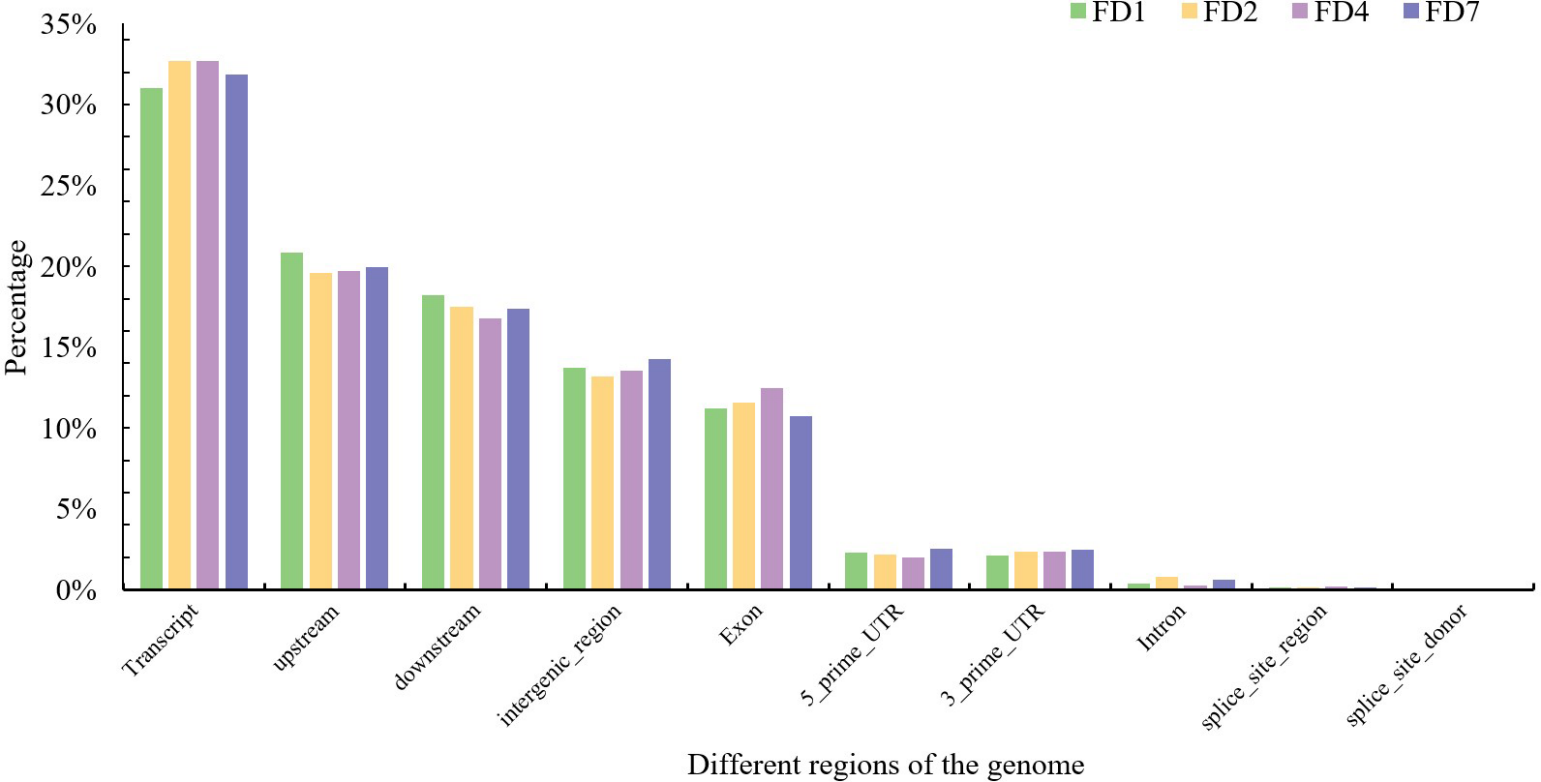
Distribution of SNP numbers across different regions of the genome.

In the four mutant strains, chromosomal deletions, duplications, and translocations were observed, with FD7 showing the highest frequency of structural variations among the four mutants (**Table 4**). This is consistent with the results showing that the pathogenicity of FD7 is lower than that of other mutants (**Figure 3**). A statistical analysis of the number of SNPs in the resistant strains revealed t hat each strain had between 400 and 460 SNPs (**Supplementary Table S2**), with transcript variants being the most abundant, exceeding 30%. Both upstream and downstream variants accounted for more than 17%, while Econ comprised about 12%, and intronic variants represented around 1% (**Figure 5**). The SNPs located within the coding regions of genes (coding SNPs, cSNPs) are particularly important in the study of genetic diseases. The results showed that: 21 genes were commonly annotated in all 4 mutants, 9 genes were commonly annotated in 3 mutants, 8 genes were commonly annotated in 2 mutants, and there were 5 genes with parental polymorphic variations (**Supplementary Table S3**).

**Table 4:** Chromosome structural variation results of drug-resistant strains.

Sample	Deletion	Duplication	Translocation	Inversion
FD1	287	77	6600	0
FD2	275	63	6596	0
FD4	190	41	3998	0
FD7	2193	1713	20120	2

### 3.5 Transcriptome changes of *B. cinerea* under TDDE treatment

**Figure 6:**
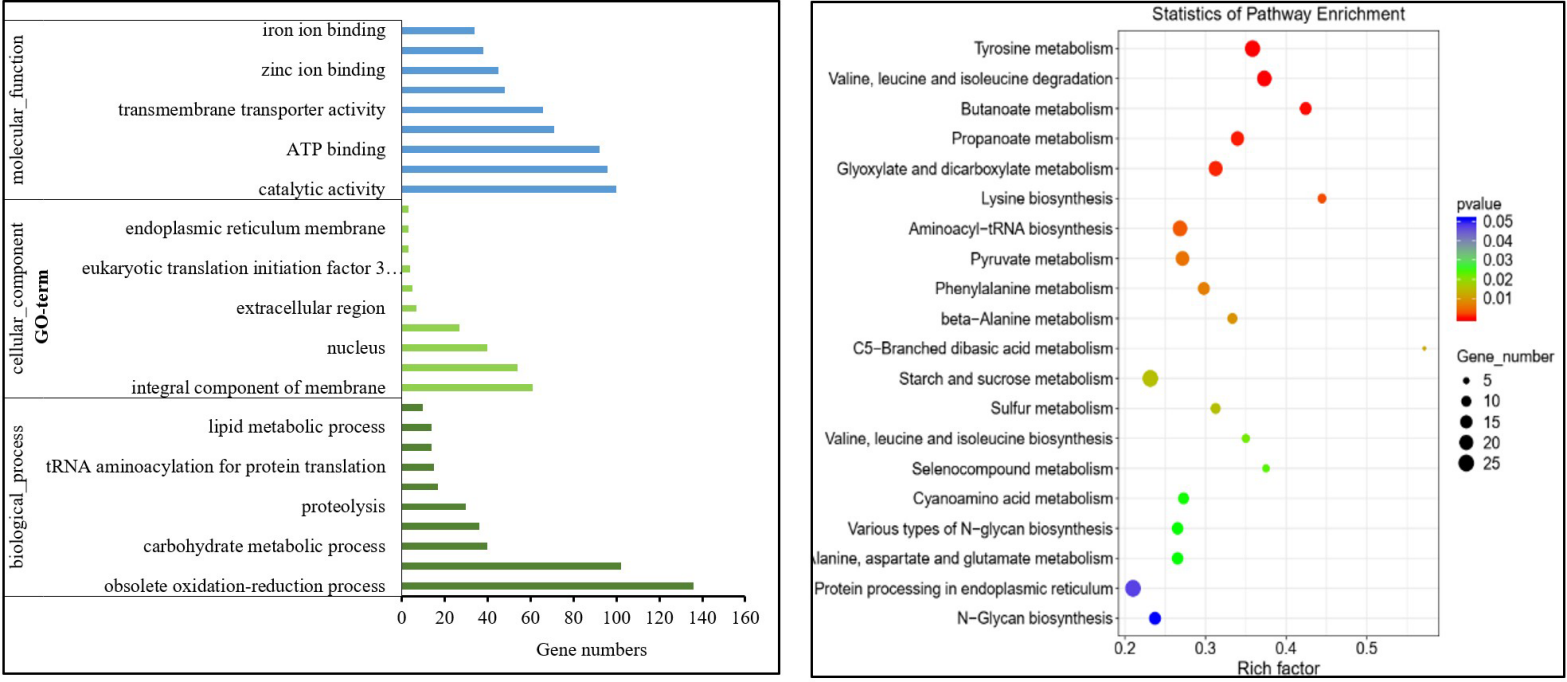
Histogram of GO enrichment and KEGG pathway enrichment for differentially expressed genes. The size of the dots represents the number of differentially expressed genes enriched in each KEGG pathway. The color of the dots indicates different P-values. The Rich factor represents the ratio of differentially expressed genes to the total number of genes in a given KEGG pathway. A larger Rich factor indicates a higher degree of enrichment for that KEGG pathway.

The experiment used a threshold of fold change (FC) ≥ 2 or FC ≤ 0.5 with a p-value< 0.05 to screen for differentially expressed genes(DEGs). The transcriptomic analysis identified 937 upregulated and 808 downregulated DEGs, with the upregulated genes exhibiting more pronounced fold-change differences compared to the downregulated genes (**Supplementary Figure S3**). The expression levels of these DEGs showed minimal variation across biological replicates, indicating high data reliability suitable for subsequent functional enrichment analysis (**Supplementary Figure S3**). Based on the number of DEGs annotated by GO, we selected the top 10 entries for analysis and visualization. The most enriched biological processes were primarily associated with redox reactions and membrane transport. Cellular components were predominantly related to membranes, while molecular functions focused on catalysis, protein binding, ATP binding, oxidoreductase activity, and transmembrane transport. TDDE treatment primarily affected genes related to membrane components, implicating them in oxidation-reduction reactions and membrane transport processes. The KEGG pathway analysis selected the top 20 most significantly enriched pathways (with smallest p-values) for scatter plot visualization, among which lysine biosynthesis showed the most prominent alterations. Other critical pathways included propionic acid metabolism, leucine and isoleucine degradation, and valine metabolism, all involving numerous DEGs (**Figure 6**). GO and KEGG analyses suggest that the DEGs are enriched in amino acid and energy metabolism, oxidase activity, and membrane lipid metabolism, potentially linking these processes to TDDE’s antifungal mechanism.

**Figure 7:**
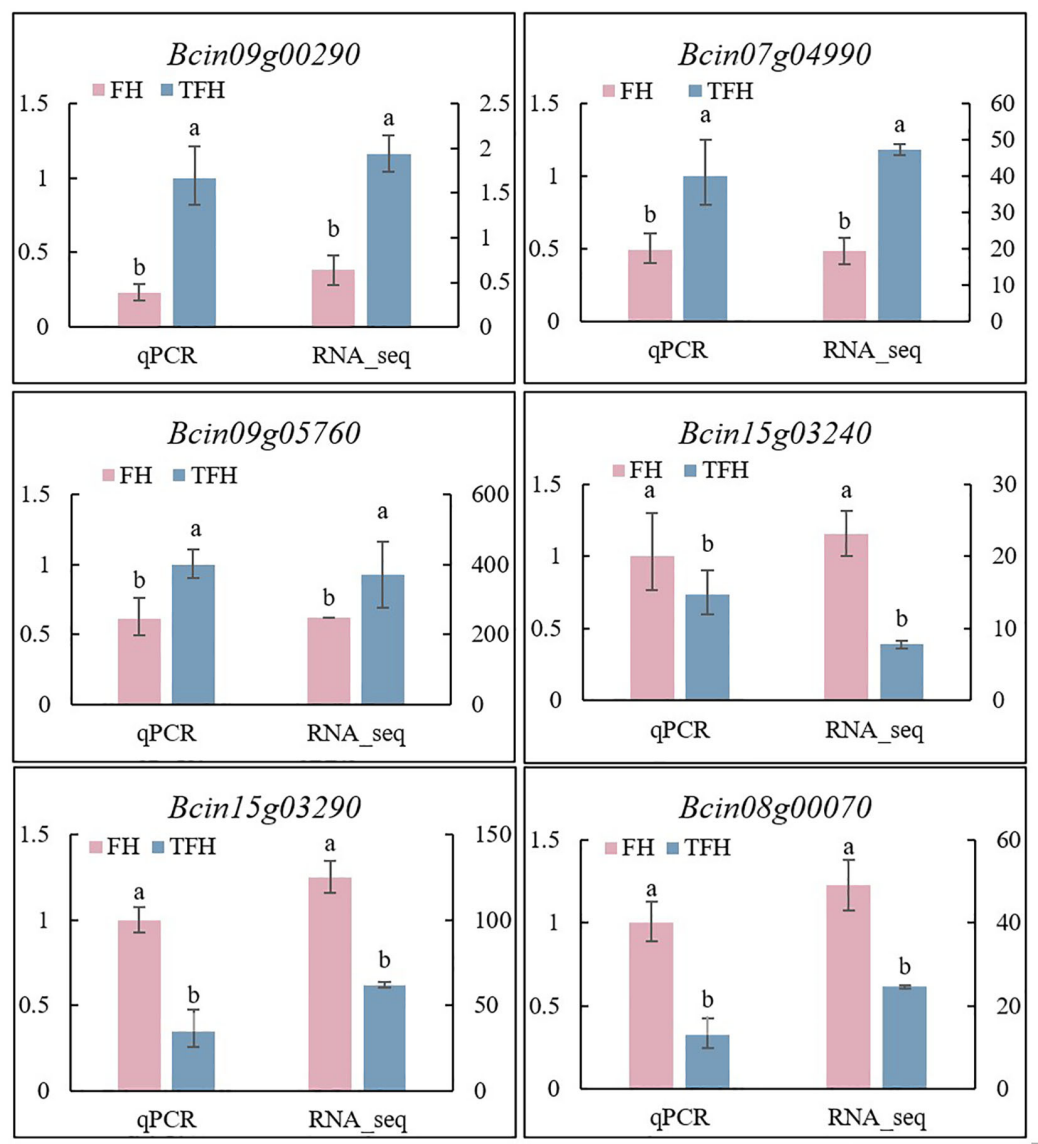
Gene relative expression levels in wild type under TDDE treatments. FH: wild type of *B*. *cinerea* (parent). TFH: wild type with 2 μg/mL TDDE treatment. qPCR: quantitative reverse transcription polymerase chain reaction. RNA_seq: mRNA sequencing. Different lowercase letters above bars indicate significant differences among treatments (p < 0.05).

The DEGs in the transcriptome were compared with the nonsynonymous cSNPs identified in the resequencing data. Six common DEGs—*Bcin15g03240*, *Bcin15g03290*, *Bcin08g00070*, *Bcin09g00290*, *Bcin07g04990*, and *Bcin09g05760*—were identified as candidate genes (**Table 5**, **Supplementary Table S4**). Both *Bcin15g03240* and *Bcin09g00290* are involved in membrane transport. These findings align with the observed changes in membrane permeability and osmotic sensitivity in the resistant strains, warranting further investigation (**Supplementary Table S4**). To validate the transcriptome data, six key genes were selected for qRT-PCR analysis. The results showed that the expression changes of three downregulated and three upregulated genes were consistent with the transcriptome data, confirming the accuracy and reliability of the findings (**Figure 7**). Additionally, the expression levels of these DEGs were significantly altered by TDDE treatment, indicating a strong response to the compound and suggesting that these genes may play a critical role in TDDE’s mechanism of action (**Figure 7**).

**Table 5:** Mutation site annotation of cSNP of target gene.

Chromosome	Strand	Start	End	Number of exons	Length	Gene ID	Gene name
chr15	+	1131808	1136350	3	4417	*Bcin15g03240*	*Bcin15g03240*
chr15	+	1151345	1154603	5	3004	*Bcin15g03290*	*Bcabp1*
chr8	+	28309	32417	10	3527	*Bcin08g00070*	*Bcin08g00070*
chr9	–	118772	122190	4	3191	*Bcin09g00290*	*Bcin09g00290*
chr7	–	1800159	1801432	2	1209	*Bcin07g04990*	*Bcin07g04990*
chr9	–	2019376	2022075	5	2204	*Bcin09g05760*	*Bcef1a*

### 3.6 The regulatory effect verification of TDDE on potential target protein

**Figure 8:**
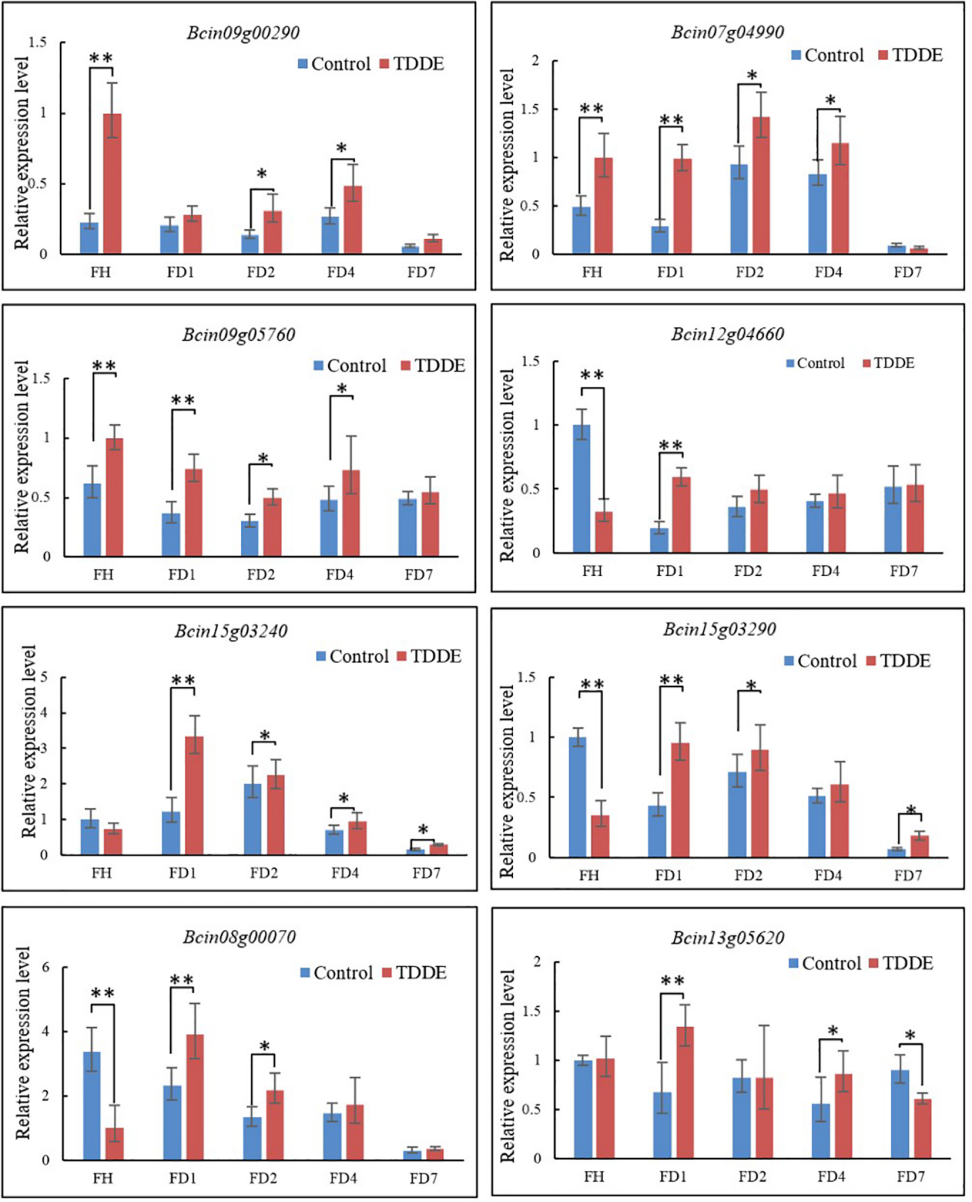
Target gene relative expression levels in wild type FH(*B. cinerea*) and mutants FD1, FD2, FD3 and FD4 under TDDE treatments, three up-regulated genes*Bcin09g00290*, *Bcin07g04990*, and *Bcin09g05760* and one non-target gene *Bcin13g04660*; three down-regulated genes *Bcin15g03240*, *Bcin15g03290*, and *Bcin08g00070* and one non-target/non-differentially expressed gene *Bcin13g05620*. The symbols represent statistical significance: * p<0.05, ** p<0.01.

To examine the regulatory effects of TDDE on the expression of six target genes, *B. cinerea* was treated with 2 μg/mL of TDDE. Fluorescence qRT-PCR was subsequently used to analyze the expression levels of six potential target genes and two non-target genes under both control and TDDE treatments. The results indicated significant differences in the expression levels of target genes in TDDE-treated strains compared to the controls (**Figure 8**), suggesting that TDDE regulates these genes’ expression in *B. cinerea*. The qRT-PCR results were consistent with transcriptome sequencing data, further confirming the accuracy of the transcriptome analysis. After TDDE treatment, the expression of candidate target genes significantly changed in the wild-type strain, but the response in TDDE-resistant mutant strains remains unclear. Therefore, the relative expression of upregulated, downregulated, and non-differentially expressed genes was measured before and after TDDE treatment. The results showed the following: ① Three upregulated genes (*Bcin09g00290*, *Bcin07g04990*, and *Bcin09g05760*) were significantly upregulated in the wild-type strain, but this upregulation was not observed in the mutant strains, particularly *Bcin09g00290*, which was upregulated approximately 4-fold in the parental strain but only 0.5 to 1-fold in the mutants. Concurrently, the expression levels of the non-target downregulated gene *Bcin12g04660* showed no significant alterations in TDDE-treated mutants compared to untreated controls, with the exception of FD1, indicating its likely non-responsiveness to TDDE treatment.② Three downregulated target genes (*Bcin15g03240*, *Bcin15g03290*, and *Bcin08g00070*) were no longer downregulated in the mutant strains, with *Bcin15g03240* showing the most significant difference. The non-target, non-differentially expressed gene *Bcin13g05620* showed no significant changes in expression levels between TDDE-treated and untreated samples across both wild-type and mutant strains, demonstrating the high accuracy of our experimental procedures (**Figure 8**). These results indicate a shift in the mutant strains’ response to TDDE, confirming the role of these target genes in the compound’s mechanism of action. In summary, the expression levels of candidate target genes in mutants, whether upregulated or downregulated, significantly differ compared to those in the wild-type FH strain. Additionally, the response of two types of non-target genes to TDDE was significantly different from that of the target genes.

### 3.7 Prediction of TDDE target protein in *B. cinerea*

**Figure 9:**
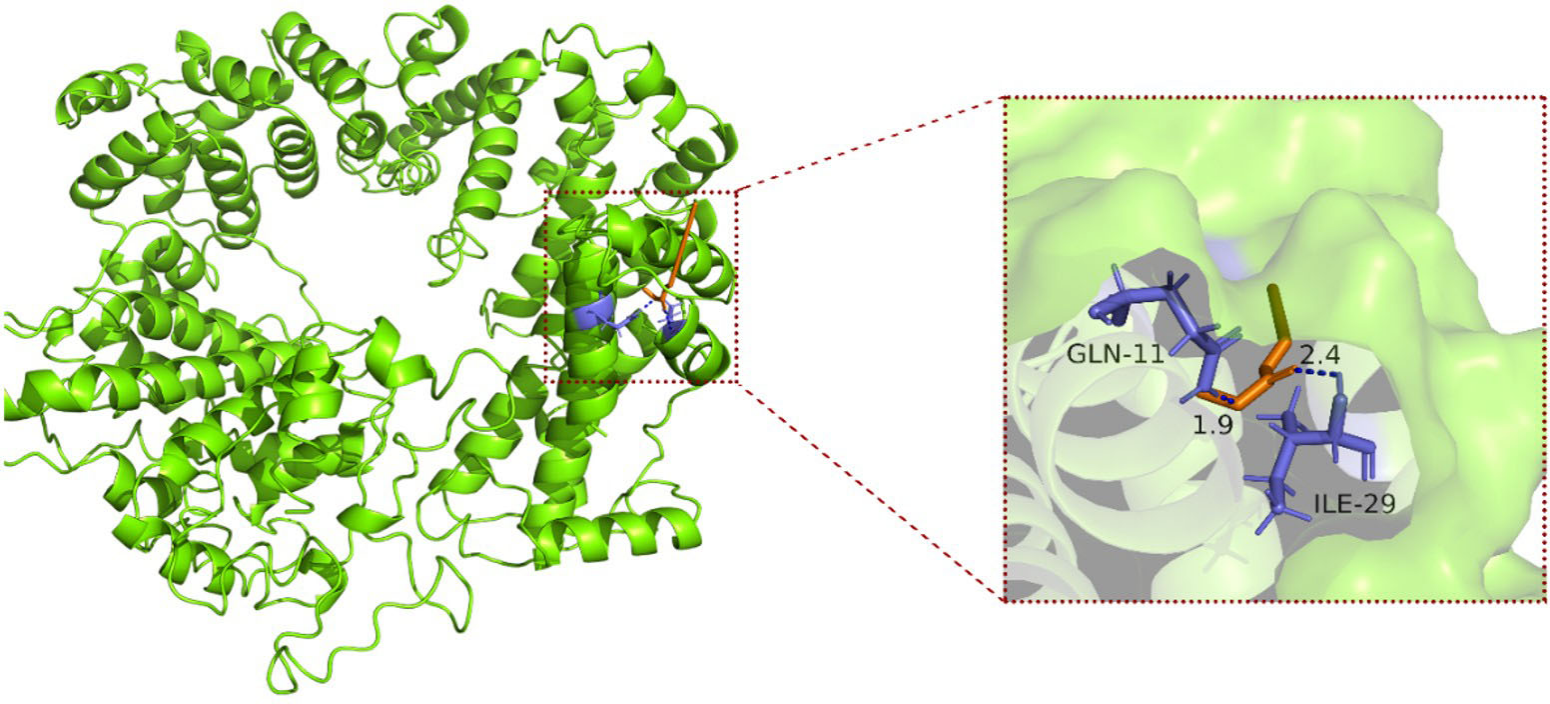
Macromolecular and enlarged local docking of TDDE and protein model molecules encoded by target gene *Bcin15g03240x*.

Based on the amino acid sequence of the target protein, we identified 10 similar template structures for each. Using sequence alignment results between the target protein and template proteins, we constructed three-dimensional structural models of the target protein through the SWISS-MODEL program. The generated models were then evaluated, and the optimal 3D structure was selected based on the comprehensive assessment presented in **Supplementary Figure S4** (**Supplementary Figure S4**). Finally, molecular docking results between TDDE and the target protein were analyzed using AutoDockTools and PyMOL software. Molecular docking analysis between TDDE and target proteins identified *Bcin15g03240* as having the lowest binding energy (-5.68 kcal/mol) with TDDE, suggesting a stable ligand-protein interaction. The protein encoded by *Bcin09g00290* also exhibited lower binding energy compared to other target proteins (**Supplementary Table S5**). Further analysis using PyMOL software demonstrated that TDDE interacted with GLN and ILE residues on the *Bcin15g03240* protein via hydrogen bonding (**Figure 9**), an interaction that implies high specificity due to the complementary chemical nature of these residues and the geometrically constrained hydrogen bonds formed with GLN. Notably, the candidate target *Bcin15g03240* was functionally annotated as a plasma membrane-localized cation transporter with specific potassium transport activity (**Supplementary Table S4**). Ortholog analysis further confirmed its high conservation as a potassium transporter across multiple fungal species, including Saccharomyces cerevisiae and Aspergillus nidulans (**Supplementary Table S7**).

## 4 Discussion

*B. cinerea*, the causal agent of tomato gray mold, is notable for its high sporulation capacity, rapid reproduction, and strong adaptability, making it a high-risk fungal pathogen for the development of resistance. This pathogen has demonstrated a strong propensity to develop resistance not only to chemical fungicides but also to botanical pesticides (Leroux et al., 1999; Esterio et al., 2007). For example, while cyprodinil initially exhibited significant inhibitory effects, its efficacy rapidly declined, with the resistance frequency of *B. cinerea* in Chile reaching 38.5% after just two years of application (Latorre et al., 2002). Cross-resistance testing, which evaluates a pathogen’s sensitivity to multiple fungicides, plays a crucial role in guiding rational fungicide use and determining whether different fungicides share similar modes of action (Mao et al., 2020). In the present study, *in vitro* bioassays revealed that TDDE possesses potent inhibitory activity against *B. cinerea*. Notably, no cross-resistance was observed between TDDE and commonly used fungicides such as pyraclostrobin, boscalid, and fludioxonil, suggesting that TDDE likely operates via a distinct mechanism of action. Furthermore, a significant negative cross-resistance was detected between TDDE and procymidone, indicating that TDDE may enhance *B. cinerea’s* sensitivity to procymidone. These findings support the potential of TDDE as a lead compound for developing novel fungicides. Its compatibility with existing fungicides makes it a promising candidate for use in combination or rotation strategies to improve control efficacy and delay resistance development in field applications.

Identifying resistance targets is essential for overcoming the challenge of fungicide resistance. The generation of resistant strains not only aids in pinpointing resistance loci but also facilitates the investigation of molecular target mechanisms, thereby laying a solid foundation for overcoming resistance and enabling the rational design of new active compounds. Research on biological characteristics such as growth rate, spore germination, sporulation, and pathogenicity provides critical information for the identification of resistant strains and the evaluation of their resistance profiles (Feng et al., 2020). In this study, resistance adaptation led to the successful selection of 52 strains with high resistance to TDDE, which were subsequently subjected to virulence assays. Based on the results, seven strains exhibiting the highest levels of resistance were selected for further characterization. Stability assays demonstrated that the resistance phenotype in these strains was stably inherited over successive generations. It is widely recognized that resistance is often associated with a reduction in biological fitness. For instance, *B. cinerea* mutants resistant to propamidine display reduced growth rates, spore germination, pathogenicity, and oxalic acid production compared to the wild-type strain (Zhang et al., 2022). We analyzed the biological characteristics of the seven highly resistant TDDE strains. The results revealed notable morphological changes, including increased branching of mycelia, which became thicker and shorter in length. Two strains exhibited slower growth rates, which was associated with reduced mycelial dry weight and sporulation. Pathogenicity tests showed that four of the resistant strains had significantly decreased virulence, and these strains were subsequently selected for physiological index measurements. Notably, no significant changes were observed in the optimal pH, temperature, or light conditions required for growth. Overall, these findings provide important insights into the biological characteristics of TDDE-resistant strains and offer valuable evidence for the further identification of resistance-associated targets and mechanisms.

Based on the integrated analysis of physiological, biochemical, and transcriptomic data, the resistance mechanism of *B. cinerea* to TDDE appears to involve multiple cellular and metabolic adaptations. Glycerol is a key osmolyte in fungal stress adaptation (Yaakoub et al., 2022); its significant reduction in resistant strains suggests a compromised ability to maintain osmotic homeostasis, thereby increasing sensitivity to environmental stress and potentially imposing a fitness cost. In this study, resistant strains exhibited enhanced sensitivity to Na^+^-induced osmotic stress and significantly reduced glycerol content, indicative of impaired osmotic regulation. This is consistent with their reduced mycelial dry weight and growth rate, suggesting that resistance acquisition is accompanied by trade-offs in growth and adaptability. In addition, resistant strains showed significantly elevated malondialdehyde (MDA) levels and relative conductivity. MDA, a byproduct of intracellular lipid peroxidation, serves as a reliable indicator of oxidative stress and membrane damage (Janero, 1990). These results point to enhanced lipid peroxidation and compromised membrane integrity in resistant strains. Supporting this, transcriptomic analyses revealed that DEGs were significantly enriched in membrane lipid metabolism, redox processes, and transmembrane transport, suggesting that TDDE disrupts membrane structure and function, triggering cellular compensatory responses. Furthermore, a marked increase in oxalic acid production was observed in resistant strains. Oxalic acid plays an important role in fungal pathogenicity and development (Nakajima and Akutsu, 2014), and its elevated levels may represent a compensatory mechanism for maintaining virulence and mitigating TDDE-induced stress through enhanced acidification and metal ion chelation (van Kan, 2006). However, excessive oxalic acid production may also impose a metabolic burden, contributing to growth retardation observed in the resistant strains. Energy metabolism was also found to be significantly altered. Resistant strains exhibited increased ATP content and ATPase activity, suggesting potentially enhanced energy generation and more active membrane transport processes. Transcriptomic data further supported this finding, with enrichment of DEGs in ATP synthesis, oxidase activity, and energy metabolism pathways. The increase in ATP levels may provide the energy required for active transport processes. In accordance with a previously proposed mechanism for fungicide resistance (Chitsaz and Brown, 2017), such ATP-dependent transport could involve efflux pumps, including ABC and MFS transporters, which have been hypothesized to reduce intracellular fungicide accumulation by expelling compounds from the cell. Metabolic reprogramming also appeared to play a critical role in resistance. Resistant strains showed significantly increased total sugar and protein content, suggesting a shift in carbon and nitrogen metabolism in response to TDDE exposure. KEGG pathway analysis revealed enrichment of DEGs involved in amino acid metabolism, including lysine biosynthesis, propionic acid metabolism, and the catabolism of branched-chain amino acids such as leucine, isoleucine, and valine. These metabolic adjustments are hypothesized to support cellular homeostasis under fungicidal stress and contribute to the survival of resistant strains. In summary, TDDE resistance in *B. cinerea* involves a complex network of adaptive changes, including altered membrane lipid metabolism, secondary metabolite biosynthesis, changes in energy metabolism, and stress response mechanisms. The observed increase in membrane permeability, oxalic acid production, ATP synthesis, oxidase activity, and amino acid metabolism highlights a multifaceted defense strategy employed by resistant strains. Nevertheless, these adaptations appear to come at a physiological cost, as evidenced by increased oxidative damage, impaired osmotic regulation, and reduced growth performance.

Mutations in target genes are widely recognized as a major mechanism underlying high-level resistance of pathogens to fungicides (Nottensteiner et al., 2019; Chen et al., 2020). *B. cinerea* has a relatively compact genome of approximately 42.65 Mb, comprising an estimated 11,700 predicted genes (Amselem et al., 2011; Staats and van Kan, 2012; Van Kan et al., 2017). Due to its manageable genome size and gene content, the integration of whole-genome resequencing with transcriptomic analysis offers a powerful strategy for identifying resistance-associated mutations and potential fungicide targets. In this study, whole-genome resequencing was conducted on four highly resistant mutant strains. The average Q30 scores exceeded 90%, indicating high sequencing quality and data reliability. A considerable number of chromosomal structural variations were detected, with the FD7 strain exhibiting the highest frequency of genomic changes, consistent with its distinct biological characteristics. Across all four mutant strains, a total of 20 single nucleotide polymorphism (SNP) genes were commonly identified. Since all four strains exhibited decreased susceptibility to TDDE, these 20 shared mutated genes were selected as key candidate target genes for further investigation. To refine this candidate list, transcriptomic analysis was employed to evaluate gene expression profiles before and after TDDE treatment, enabling the identification of DEGs potentially involved in TDDE response. By integrating the transcriptomic and genomic datasets, six overlapping genes were identified: *Bcin15g03240*, *Bcin15g03290*, *Bcin08g00070*, *Bcin09g00290*, *Bcin07g04990*, and *Bcin09g05760*. Studies have shown that the *Bcin08g00070* gene encodes a cytoplasmic glycosyltransferase GT28, which plays an important role in helping *Botrytis cinerea* counteract the toxicity of plant saponins by participating in membrane repair mechanisms (You et al., 2024). This finding further supports the possibility that these candidate genes may indeed be involved in conferring resistance to TDDE. These genes were considered as primary candidate targets for subsequent functional validation. It is important to acknowledge potential limitations in this approach. For instance, genes uniquely mutated in the FD7 strain—which exhibited the most pronounced resistance phenotype—were excluded from the list of common mutations and, therefore, from further consideration. Despite their possible critical roles in resistance development, such strain-specific mutations may have been overlooked due to the focus on mutations shared across all four strains.

Molecular docking is a widely used technique for assessing the binding affinity and interaction strength between small molecules and target proteins (Fan et al., 2019). For instance, a network pharmacology and molecular docking study revealed that arctiin and linarin, the core components of Jinhua Qinggan Granules (JQG), exhibited high binding affinities to key targets including COVID-19 3CL hydrolase and ACE2, suggesting a common mechanism of JQG in treating coronavirus diseases such as SARS, MERS, and COVID-19 (Zhang et al., 2021). In the present study, molecular docking was performed between the TDDE molecule and the six candidate target proteins. The results demonstrated a particularly strong interaction between TDDE and the protein encoded by *Bcin15g03240*, indicating that this gene may serve as a primary molecular target of TDDE. In addition, all six target proteins exhibited notable binding affinities with TDDE, suggesting that TDDE may exert its antifungal activity through a multi-target mechanism. These findings not only support the potential role of *Bcin15g03240* in mediating TDDE resistance but also highlight the broader molecular basis of TDDE’s antifungal action.

In conclusion, this study demonstrates that TDDE is a novel lead compound with a distinct mode of action. It can be used in tank mixtures or alternations with pyraclostrobin, boscalid, procymidone, and fluazinam under field conditions. Resistance to TDDE in *B. cinerea* is associated with multifaceted adaptive costs, including cellular membrane damage, impaired osmotic regulation, oxidative stress, and metabolic reprogramming, potentially linked to multiple genetic mutations. Finally, by combining whole-genome resequencing with transcriptomic data, we refined the list of resistance-associated candidate target genes from 20 to 6. Molecular docking confirmed a strong binding affinity between TDDE and the protein encoded by *Bcin15g03240*, suggesting it as a potential primary target. These findings provide a solid foundation for developing new fungicides and designing resistance management strategies.

## Data Availability

The datasets presented in this study can be found in online repositories. The names of the repository/repositories and accession number(s) can be found in the article/**Supplementary Material**.
