# Correction: Biochemical and genetic characterization of *Botrytis cinerea* mutants resistant to the plant-derived pesticide trans-dehydromatricaria ester

**DOI:** 10.3389/fpls.2025.1760314

**Published:** 2026-01-19

**Authors:** Gaijuan Tang, Rui Nan, Hui Dong, Yong Wang, Wenhui Guo, Yongquan Ta, Yunfei Han, Chao Zhang, Yonghong Wang

**Affiliations:** 1 Northwest Agriculture and Forestry (A&F) University, Yangling, Shaanxi, China; 2 Hybrid Rapeseed Research Center of Shaanxi Province, Yangling, Shaanxi, China; 3 College of Agriculture, Ningxia University, Yinchuan, Ningxia, China; 4 Environmental and Plant Protection Institute, Chinese Academy of Tropical Agricultural Sciences, Haikou, Hainan, China

**Keywords:** *Botrytis cinerea*, trans-dehydromatricaria ester (TDDE), drug resistance, antibacterial mechanism, molecular targets

Author Yunfei Han was erroneously spelled as Y. F. Han.

The original version of this article has been updated.

An erroneous citation callout [13] was included in the **Introduction**, Paragraph 2:

“Artemisia ordosica, a wild plant with multiple potential applications, contains the bioactive compound TDDE, which has demonstrated broad-spectrum antifungal activity and holds promise as a novel fungicide.”

A space was missing in the temperature notation.

A correction has been made to section 2.11, second sentence:

The plates were incubated at 25 °C in a constant-temperature chamber for 3 days.

There was a mistake in **Figure 4G** as published. The y-axis label was misspelled as “Ttotal protein content (mg/g)”. The corrected **Figure 4G** appears below.

**Figure 4:**
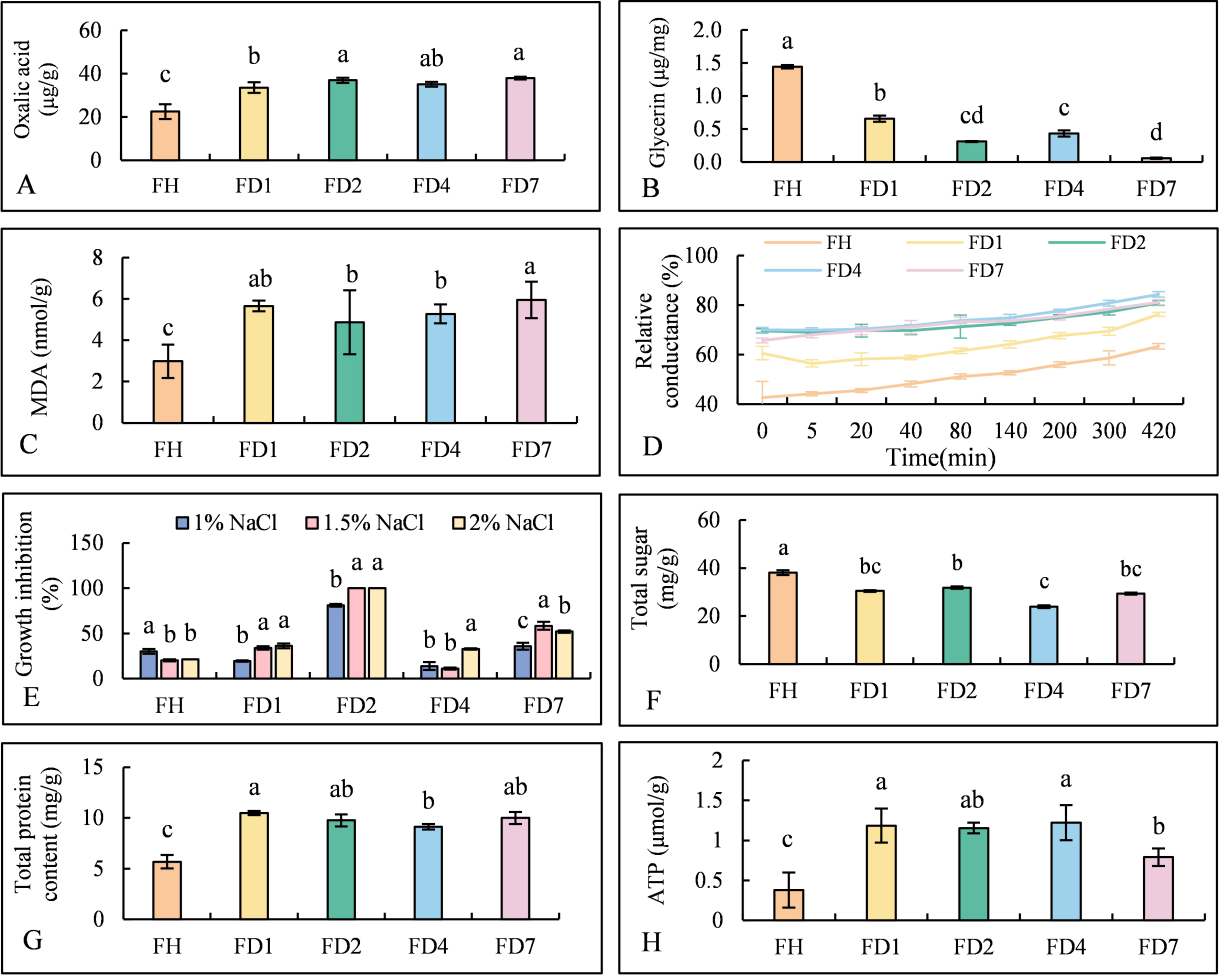
The differences in physiological characteristics between the parental of B. cinerea Strains and the drug resistant strains. **(A)** Oxalic Acid Content. **(B)** Glycerin. **(C)** MDA. **(D)** Relative Conductivity. **(E)** NaCl Sensitivity. **(F)** Total Sugar. **(G)** total protein content. **(H)** ATPase Activity. Different lowercase letters above bars indicate significant differences among treatments (p < 0.05).

The original version of this article has been updated.

The symbol for the correlation coefficient was incorrect n section 2.6, the last sentence:

“According to the correlation coefficient (ρ), cross-resistance was categorized as follows: no cross-resistance (ρ > 0.05 or ρ < 0.1), significant cross-resistance (0.8 < ρ ≤ 1 and ρ < 0.05), strong cross-resistance (0.6 ≤ ρ ≤ 0.8), moderate cross-resistance (0.4 ≤ ρ < 0.6), low cross-resistance (0.2 ≤ ρ < 0.4), and very low cross-resistance (0.1 ≤ ρ < 0.2).”

The citation format in section 2.6 was incomplete at the end of the paragraph:

The citation: (Li, 2021) has been corrected to: (Li, X. H., 2021).

The original version of this article has been updated.

