# Supplementary material for: Biochemical and genetic characterization of Botrytis cinerea mutants resistant to the plant-derived pesticide trans-dehydromatricaria ester
Source: Front Plant Sci. 2025 Nov 20;16:1715720. doi: 10.3389/fpls.2025.1715720 (PMC12676266; doi:10.3389/fpls.2025.1715720)
Supplement: Supplementary file 1 [file DataSheet1.docx]

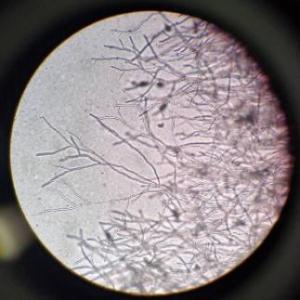

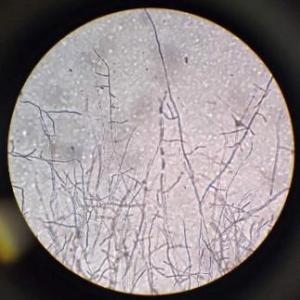

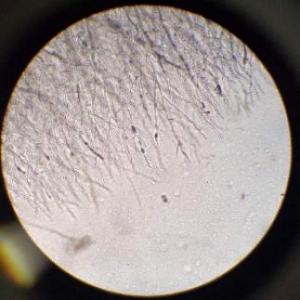

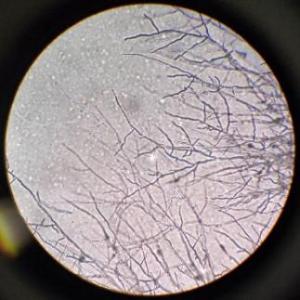

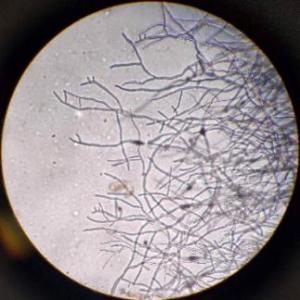

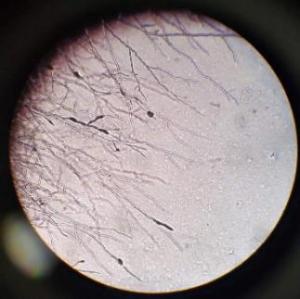

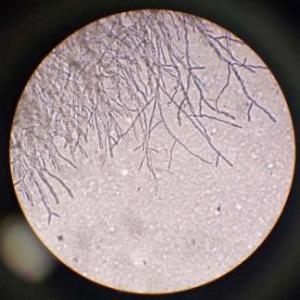

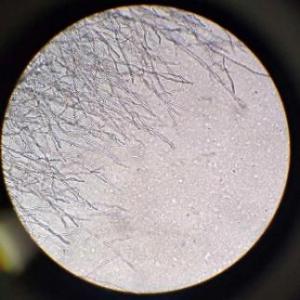


FH

FD1

FD2

FD6

FD7

FD3

FD4

FD5

Figure S1 Observation of mycelial morphology difference between the drug-resistant strains and their parents under ordinary light microscope

Note: FH is the parent. FD1 to FD7 were resistant strains. Observations were made at 10 magnification.


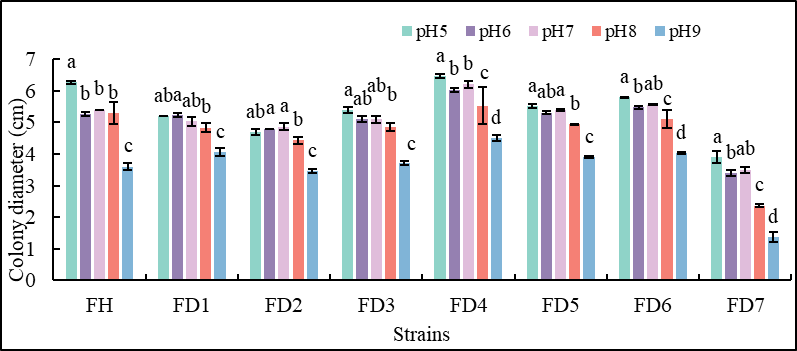

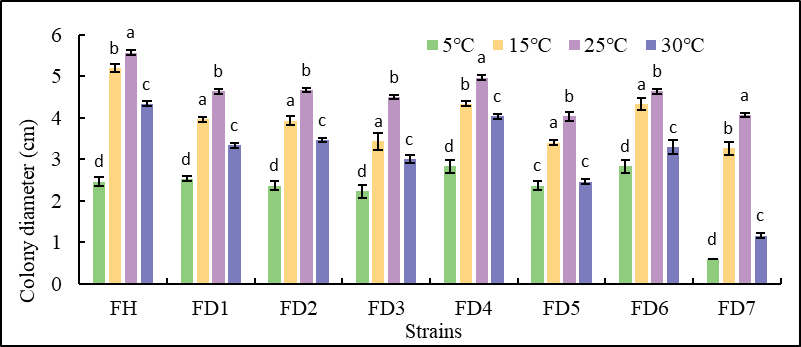

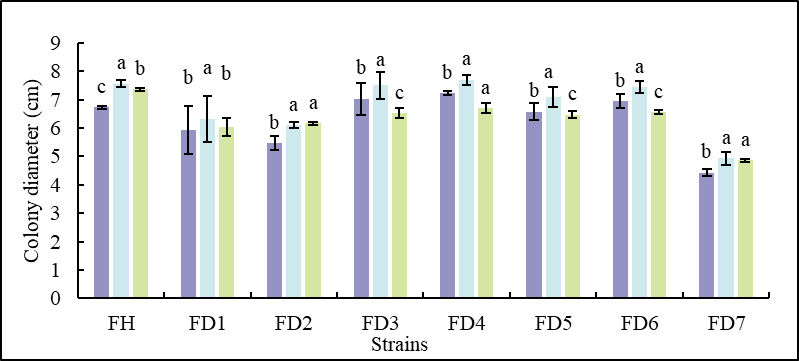


0h light exposure


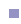


12h light exposure


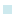


24h light exposure


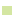


Figure S2 Effects of different pH, temperature and illumination on mycelial growth of resistance strains

Note: Different lowercase letters in the figures indicate signiﬁcant differences among different strains at 0.05 level.


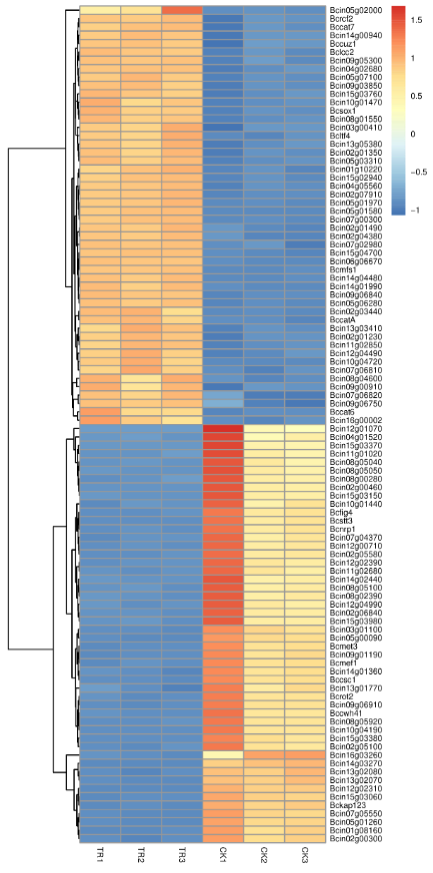

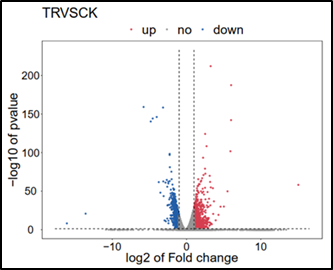


C

B


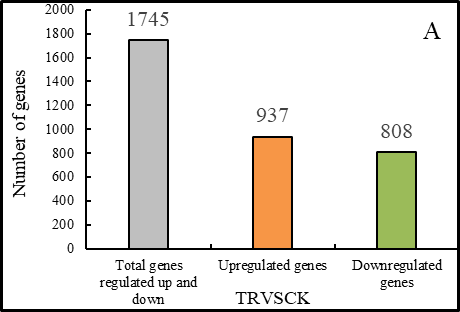


Figure S3 Histogram, volcano diagram and heat map of the statistical number of differentially expressed genes.

Note: A is the histogram of differentially expressed genes, red represents significantly differentially expressed up-regulated genes, blue represents significantly differentially expressed down-regulated genes, and gray represents the total number of differentially expressed genes. B is the volcanic map of differential genes, with log2(fold change) as abscissa and -log10(P value) as ordinate. The gray dots represent non-significant genes. C is heat map of differential genes. Red represents up-regulated genes, blue represents down-regulated genes.

Figure S4 Construction of 3D models of target gene proteins.


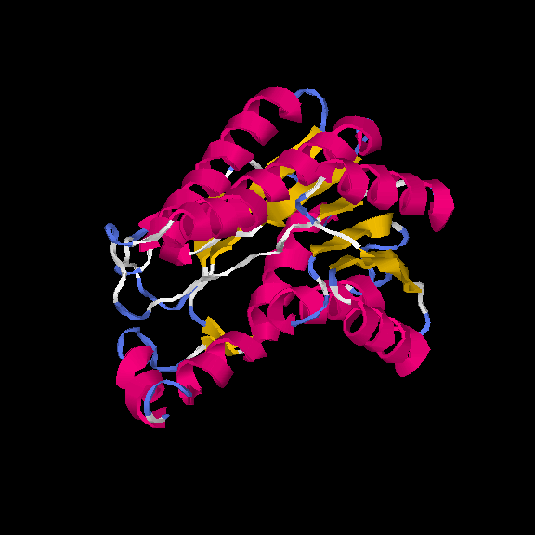

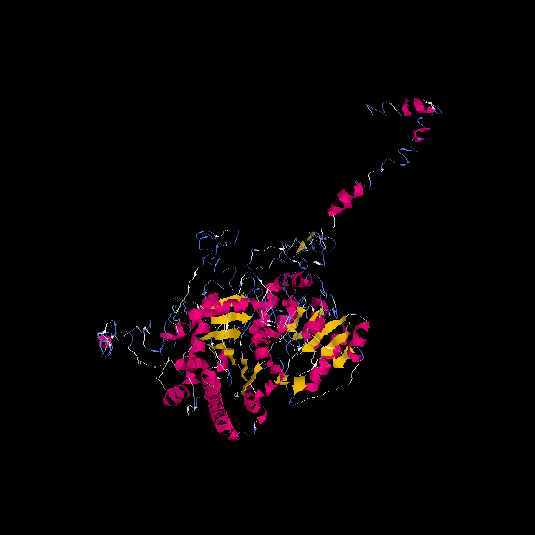

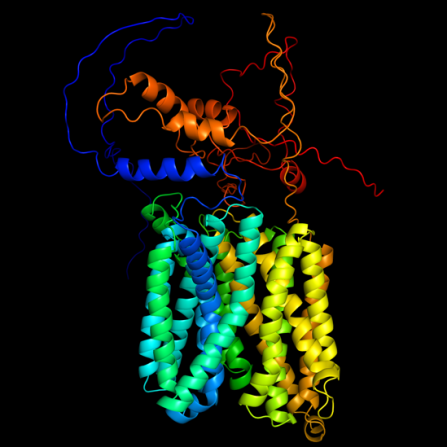

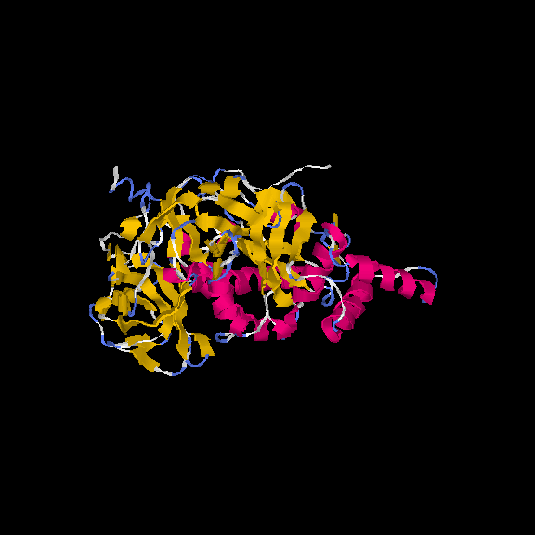

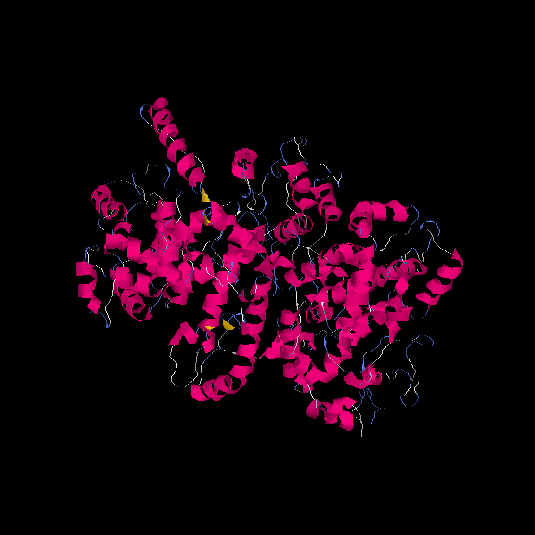

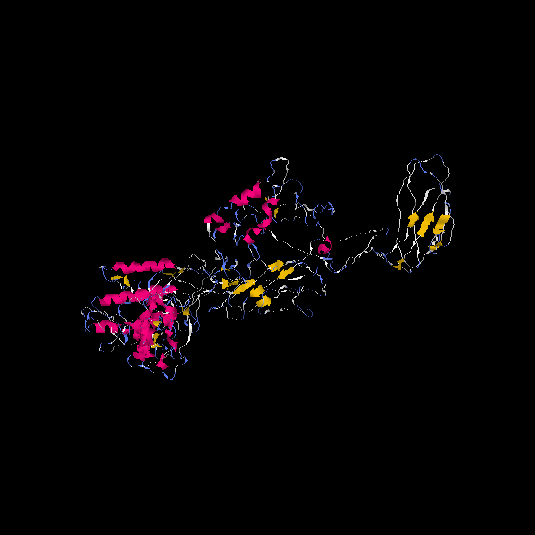


*Bcin15g03240*

*Bcin15g03290*

*Bcin08g00070*

*Bcin09g05760*

*Bcin07g04990*

*Bcin09g00290*

Table S1 Pathogenicity assessment of parental and TDDE-resistant mutant strains on different host tissues.

| Plant | Stains | Parent | FD1 | FD2 | FD4 | FD7 |
| --- | --- | --- | --- | --- | --- | --- |
| Strawberry leaves | Lesion Area (mm²) | 585.83±54.93 | 126.88±76.41*** | 323.36±46.93** | 224.75±71.61*** | 38.44±16.9*** |
|  | Relative Lesion Area (%) | 8.26±0.77 | 1.46±0.88*** | 4.16±0.6** | 2.55±0.81*** | 0.29±0.13*** |
| Tomato leaves | Lesion Area (mm²) | 395.86±35.25 | 70.07±8.05*** | 144.17±25.33*** | 101.79±10.76*** | 27.67±1.41*** |
|  | Relative Lesion Area (%) | 30.11±2.68 | 5.97±0.69*** | 13.73±2.41*** | 7.53±0.8*** | 2.22±0.11*** |
| Tomato seedling | Lesion Area (mm²) | 702.87±35.68 | 33.51±0.5*** | 117.2±15.12*** | 265.11±24.07*** | 27±0.92*** |
|  | Relative Lesion Area (%) | 30.6±1.55 | 1.8±0.03*** | 5.07±0.65*** | 14.44±1.31*** | 1.93±0.07*** |
| Note: Data are presented as mean ± SD. Asterisks indicate significant differences compared to the parental strain as determined by Tukey's test (*p ≤ 0.05, **p ≤ 0.01, ***p ≤ 0.001). Relative Lesion Area (%) was calculated as (Lesion Area / Total Leaf Area) × 100%. | | | | | | |

Table S2 SNP statistical results of drug-resistant strains

| Sample | SNP number | Transition | Transvers | Ti/Tv | Heterozygosity | Homozygosity | Het-ratio |
| --- | --- | --- | --- | --- | --- | --- | --- |
| FD1 | 448 | 347 | 101 | 3.43 | 416 | 32 | 92.85% |
| FD2 | 408 | 321 | 87 | 3.68 | 377 | 31 | 92.4% |
| FD4 | 411 | 326 | 85 | 3.83 | 388 | 23 | 94.4% |
| FD7 | 456 | 376 | 121 | 3.97 | 420 | 35 | 96.5% |

Table S3 Genes of SNP mutations were annotated in two or more mutants

| Mutant number | Gene_ID | NR_annotation | Swiss_Prot_NAME |
| --- | --- | --- | --- |
| 4 | *Bcin02g00003* | hypothetical protein BCIN_02g00003  [*Botrytis cinerea* B05.10] | - |
| 4 | *Bcin02g00004* | hypothetical protein BCIN_02g00004  [*Botrytis cinerea* B05.10] | - |
| 4 | *Bcin02g00005* | hypothetical protein BCIN_02g00005  [*Botrytis cinerea* B05.10] | - |
| 4 | *Bcin02g00008* | hypothetical protein BCIN_02g00008  [*Botrytis cinerea* B05.10] | - |
| 4 | *Bcin02g00009* | hypothetical protein BCIN_02g00009  [*Botrytis cinerea* B05.10] | - |
| 4 | *Bcin11g05880* | hypothetical protein BCIN_11g05880  [*Botrytis cinerea* B05.10] | - |
| 4 | *Bcin11g06560* | hypothetical protein BCIN_11g06560  [*Botrytis cinerea* B05.10] | - |
| 4 | *Bcin14g00610* | Bcpg2 [*Botrytis cinerea* B05.10] | PG1 |
| 4 | *Bcin14g00620* | hypothetical protein BCIN_14g00620  [*Botrytis cinerea* B05.10] | - |
| 4 | *Bcin15g00010* | hypothetical protein BCIN_15g00010  [*Botrytis cinerea* B05.10] | - |
| 4 | *Bcin15g03240* | hypothetical protein BCIN_15g03240  [*Botrytis cinerea* B05.10] | TRK2 |
| 4 | *Bcin15g03250* | hypothetical protein BCIN_15g03250  [*Botrytis cinerea* B05.10] | - |
| 4 | *Bcin15g03290* | Bcabp1 [*Botrytis cinerea* B05.10] | Dbnl |
| 4 | *Bcin15g03310* | Bcgrx3 [*Botrytis cinerea* B05.10] | GRX3 |
| 4 | *Bcin15g03410* | hypothetical protein BCIN_15g03410  [*Botrytis cinerea* B05.10] | MGA2 |
| 4 | *Bcin15g03420* | Bcpmt2 [*Botrytis cinerea* B05.10] | PMT2 |
| 4 | *Bcin15g03430* | putative high affinity glucose transporter protein [*Botrytis cinerea* BcDW1] | HGT1 |
| 4 | *Bcin15g03440* | hypothetical protein BCIN_15g03440  [*Botrytis cinerea* B05.10] | CHS7 |
| 4 | *Bcin15g03450* | Bcssl1 [*Botrytis cinerea* B05.10] | tfh47 |
| 4 | *Bcin06g05180* | hypothetical protein BCIN_06g05180  [*Botrytis cinerea* B05.10] | SPBC3E7.04c |
| 4 | *Bcin07g00730* | hypothetical protein BCIN_07g00730  [*Botrytis cinerea* B05.10] | otaA |
| 3 | *Bcin01g00140* | Bcboa15 [Botrytis cinerea B05.10] | - |
| 3 | *Bcin10g06270* | hypothetical protein BCIN_10g06270  [*Botrytis cinerea* B05.10] | - |
| 3 | *Bcin11g00720* | hypothetical protein BCIN_11g00720  [*Botrytis cinerea* B05.10] | - |
| 3 | *Bcin11g00730* | hypothetical protein BCIN_11g00730  [*Botrytis cinerea* B05.10] | - |
| 3 | *Bcin13g00790* | Bcmrpl17 [*Botrytis cinerea* B05.10] | - |
| 3 | *Bcin13g00810* | Bcdao3 [*Botrytis cinerea* B05.10] | DAO1 |
| 3 | *Bcin13g01020* | hypothetical protein BCIN_13g01020  [*Botrytis cinerea* B05.10] | - |
| 3 | *Bcin08g00070* | hypothetical protein BCIN_08g00070  [*Botrytis cinerea* B05.10] | UGT80B1 |
| 3 | *Bcin09g00290* | hypothetical protein BCIN_09g00290  [*Botrytis cinerea* B05.10] | - |
| 2 | *Bcin06g02860* | hypothetical protein BCIN_06g02860  [*Botrytis cinerea* B05.10] | tea1 |
| 2 | *Bcin06g02870* | hypothetical protein BCIN_06g02870  [*Botrytis cinerea* B05.10] | hhp1 |
| 2 | *Bcin07g04990* | hypothetical protein BCIN_07g04990  [*Botrytis cinerea* B05.10] | lvr |
| 2 | *Bcin07g05000* | hypothetical protein BCIN_07g05000 [*Botrytis cinerea* B05.10] | - |
| 2 | *Bcin09g05760* | Bcef1a [*Botrytis cinerea* B05.10] | TEF1 |
| 2 | *Bcin12g01630* | hypothetical protein BCIN_12g01630  [*Botrytis cinerea* B05.10] | - |
| 2 | *Bcin16g02970* | hypothetical protein BCIN_16g02970  [*Botrytis cinerea* B05.10] | ABCC1 |
| 2 | *Bcin16g04560* | hypothetical protein BCIN_16g04560  [*Botrytis cinerea* B05.10] | - |
| 0 | *Bcin02g00006* | hypothetical protein BCIN_02g00006  [*Botrytis cinerea* B05.10] | - |
| 0 | *Bcin02g00007* | hypothetical protein BCIN_02g00007  [*Botrytis cinerea* B05.10] | - |
| 0 | *Bcin02g06410* | Bcsgd1 [*Botrytis cinerea* B05.10] | sgd1 |
| 0 | *Bcin10g06250* | hypothetical protein BCIN_10g06250  [*Botrytis cinerea* B05.10] | - |
| 0 | *Bcin13g05150* | hypothetical protein BCIN_13g05150  [*Botrytis cinerea* B05.10] | - |

Note: "0" is the wild-type variation itself, which occurs in each mutant.

Table S4 Annotation of differentially expressed genes.

| Gene ID | *Bcin15g03240* | *Bcin15g03290* | *Bcin08g00070* | *Bcin09g00290* | *Bcin07g04990* | *Bcin09g05760* |
| --- | --- | --- | --- | --- | --- | --- |
| Gene Name | *Bcin15g03240* | *Bcabp1* | *Bcin08g00070* | *Bcin09g00290* | *Bcin07g04990* | *Bcef1a* |
| GO | GO:0005887(integral component of plasma membrane); GO:0006812(cation transport); GO:0008324(cation transmembrane transporter activity); GO:0015079(potassium ion transmembrane transporter activity); GO:0016021(integral component of membrane); GO:0030007(cellular potassium ion homeostasis); GO:0055085(transmembrane transport) | GO:0003779(actin binding); GO:0005515(protein binding) | GO:0005975(carbohydrate metabolic process); GO:0016758(transferase activity, transferring hexosyl groups); GO:0030259(lipid glycosylation) | GO:0022857(transmembrane transporter activity); GO:0055085(transmembrane transport) | GO:0016491(oxidoreductase activity) | GO:0003746(translation elongation factor activity); GO:0003924(GTPase activity); GO:0005525(GTP binding); GO:0006414(translational elongation); GO:0006414(translational elongation) |
| KEGG | NA | NA | 00970(Aminoacyl-tRNA biosynthesis) | 00300(Lysine biosynthesis) | NA | 03013(RNA transport); |
| KO_ID | NA | NA | K01870 | K01705 | NA | K03231 |
| EC | NA | NA | EC:6.1.1.5 | EC:4.2.1.36 | NA | NA |
| regulation | down | down | down | up | up | up |
| significant | yes | yes | yes | yes | yes | yes |
| NR annotation | hypothetical protein *BCIN_15g03240 [B. cinerea B05.10]* | Bcabp1 *[B. cinerea B05.10]* | hypothetical protein *BCIN_08g00070 [B. cinerea B05.10]* | hypothetical protein *BCIN_09g00290 [B. cinerea B05.10]* | hypothetical protein *BCIN_07g04990 [B. cinerea B05.10]* | Bcef1a *[B. cinerea B05.10]* |
| Swiss Prot Name | TRK2 | Dbnl | UGT80B1 |  | lvr | TEF1 |
| Protein | Low-affinity potassium transport protein; Potassium transport protein | Drebrin-like protein；Drebrin-like protein; Bcabp1 | Sterol 3-beta-glucosyltransferase UGT80B1; Glyco_transf_28 domain-containing protein | MFS domain-containing protein | Uncharacterized protein | Elongation factor 1-alpha；Elongation factor 1-alpha 1 |
| ID | P28584；Q10065 | Q62418；Q9JHL4 | Q9XIG1 |  |  | P02994；P0CY35 |
| UniProt | A0A384K4V6 | A0A384K4P4 | A0A384JNQ5 | A0A384JRL5 | A0A384JN02 | A0A384JTH0 |

Table S5 Molecular docking values of target gene proteins

| Gene_ID | Compound | Binding energy  (kcal/mol) | Ligand efficiency  (kcal/mol) | Inhibit constant  (µm) | Intermol energy  (kcal/mol) | Total energy  (kcal/mol) | Unbound  Energy  (kcal/mol) |
| --- | --- | --- | --- | --- | --- | --- | --- |
| *Bcin15g03240* | TDDE | -5.68 | -0.44 | 68.24 | -7.17 | -0.31 | -0.31 |
| *Bcin15g03290* | TDDE | -4.68 | -0.36 | 370.56 | -6.17 | -0.29 | -0.29 |
| *Bcin08g00070* | TDDE | -5.02 | -0.39 | 210.51 | -6.51 | -0.29 | -0.20 |
| *Bcin09g00290* | TDDE | -5.54 | -0.43 | 86.59 | -7.03 | -0.32 | -0.32 |
| *Bcin07g04990* | TDDE | -5.19 | -0.4 | 156.95 | -6.68 | -0.27 | -0.27 |
| *Bcin09g05760* | TDDE | -4.91 | -0.38 | 250.22 | -6.41 | -0.3 | -0.3 |

Table S6 Primers for RT-PCR

| Number | Primer Sequence | Sequence Definition | Sequence Length | | Pair Rating | | Product Length | |
| --- | --- | --- | --- | --- | --- | --- | --- | --- |
| qPCR-Bc4990-F | CGAGTGGGAATTTGTCTTGGGAGTC | Bcin07g04990.1 cds chromosome꞉ASM83294v1꞉7꞉1800159꞉1801432꞉-1 gene꞉Bcin07g04990 gene_biotype꞉protein_coding transcript_biotype꞉protein_coding | 777 | | 81.4 | | 269 |  |
| qPCR-Bc4990-R | CCGCAGTCTTGAGCATTGGTGTA |  |  |  |  |  |  |  |
| qPCR-Bc0070-F | GACCAACTTCACCCTCGGCTTTAG | Bcin08g00070.1 cds chromosome꞉ASM83294v1꞉8꞉28309꞉32417꞉1 gene꞉Bcin08g00070 gene_biotype꞉protein_coding transcript_biotype꞉protein_coding | 2,481 | | 80 | | 256 |  |
| qPCR-Bc0070-R | ACCACCACTTCTCCAATCCTTAACC |  |  |  |  |  |  |  |
| qPCR-Bc0290-F | CGCCATCGGACTCGCATGTATC | Bcin09g00290.1 cds chromosome꞉ASM83294v1꞉9꞉118772꞉122190꞉-1 gene꞉Bcin09g00290 gene_biotype꞉protein_coding transcript_biotype꞉protein_coding | 2,157 | | 82 | | 213 |  |
| qPCR-Bc0290-R | CGCTGAACTCGTTCGTCAATGTTG |  |  |  |  |  |  |  |
| qPCR-Bc5760-F | ACATGATCGACAACTCCACCAACTG | Bcin09g05760.1 cds chromosome꞉ASM83294v1꞉9꞉2019376꞉2022075꞉-1 gene꞉Bcin09g05760 gene_biotype꞉protein_coding transcript_biotype꞉protein_coding gene_symbol꞉Bcef1a | 1,383 | | 81.5 | | 192 |  |
| qPCR-Bc5760-R | GCACCGTTCCAATACCACCAATCT |  |  |  |  |  |  |  |
|  |  |  |  | |  | |  |  |
| qPCR-Bc4660-F | GCACCGTTGGCTCAAGAGAACAA | Bcin12g04660.1 cds chromosome꞉ASM83294v1꞉12꞉1581016꞉1583004꞉-1 gene꞉Bcin12g04660 gene_biotype꞉protein_coding transcript_biotype꞉protein_coding gene_symbol꞉Bctcp1 | 1,701 | | 80.7 | | 174 |  |
|  |  |  |  |  |  |  |  |  |
| qPCR-Bc4660-R | CATCTTCGTTAGCATCGCCATCCTT |  |  |  |  |  |  |  |
| qPCR-Bc5620-F | TGGACACAGTGACGAGGCAAGA | Bcin13g05620.1 cds chromosome꞉ASM83294v1꞉13꞉2141440꞉2142928꞉1 gene꞉Bcin13g05620 gene_biotype꞉protein_coding transcript_biotype꞉protein_coding gene_symbol꞉Bccyb5 | | 420 | | 81 | 161 |  |
| qPCR-Bc5620-R | GAATGATAGCGTAGAGACCGACACC |  |  |  |  |  |  |  |
| qPCR-Bc3240-F | GGCACAGTTGGTCTGAGTCTAGGA | Bcin15g03240.1 cds chromosome꞉ASM83294v1꞉15꞉1131808꞉1136350꞉1 gene꞉Bcin15g03240 gene_biotype꞉protein_coding transcript_biotype꞉protein_coding | | 2,724 | | 83.1 | 267 |  |
| qPCR-Bc3240-R | GCCAGTGTTGACAGTTGCGAGAT |  |  |  |  |  |  |  |
| qPCR-Bc3290-F | AAGTGGAGGAGGACAATGGAAGAGT | Bcin15g03290.1 cds chromosome꞉ASM83294v1꞉15꞉1151345꞉1154603꞉1 gene꞉Bcin15g03290 gene_biotype꞉protein_coding transcript_biotype꞉protein_coding gene_symbol꞉Bcabp1 | | 2,505 | | 86.8 | 268 |  |
| qPCR-Bc3290-R | GACAGGTGGAGGTGGTGGAGATT |  |  |  |  |  |  |  |
| P-33 | CGTCTGGATTGGTGGTTCTATT | - | | - | |  | - |  |
| P-34 | ACTCGTCGTACTCTTGCTTTG | - | | - | |  | - |  |

Note: P-33 and P-34 are internal reference primers

Table S7 Orthologs and functional annotation of the leading candidate target gene Bcin15g03240 in representative fungal species.

| Species | Ortholog / Description | Accession | Identity (%) | E-value | Query Cover (%) | Known/ Predicted Function |
| --- | --- | --- | --- | --- | --- | --- |
| Saccharomyces cerevisiae | Trk1p | NP_012406.1 | 53.57 | 9e-153 | 68 | High-affinity potassium transporter |
| Saccharomyces cerevisiae | Trk2p | NP_012976.1 | 40.84 | 0.0 | 86 | Potassium transporter |
| Aspergillus nidulans | protein trkA | XP_050468222.1 | 49.88 | 2e-123 | 56 | Potassium transporter |
| Magnaporthe oryzae | potassium transport protein | ELQ32851.1 | 52.70 | 6e-136 | 54 | Potassium transport |
| Neurospora crassa | potassium transporter | XP_959511.2 | 34.70 | 2e-58 | 52 | Potassium transporter |
| Note: BLASTP analysis was performed against the non-redundant protein sequences (nr) database at NCBI. The orthologs listed represent the top significant hits from searches limited to each specified organism. The candidate gene Bcin15g03240 is consistently annotated as an integral component of membrane with cation transmembrane transporter activity, and its orthologs across these diverse fungal species are all known or predicted potassium transporters, underscoring its conserved functional role. | | | | | | |
